# Supplementary material for: What does it take to learn the rules of RNA base pairing? A lot less than you may think
Source: bioRxiv. 2025 Aug 2:2025.07.31.668042. Preprint. [Version 1] doi: 10.1101/2025.07.31.668042 (PMC12324431; doi:10.1101/2025.07.31.668042)
Supplement: Supplement 1 [file media-1.gz › supplemental_material/Figures_data/Figure_4/Figure_R3.pdf]

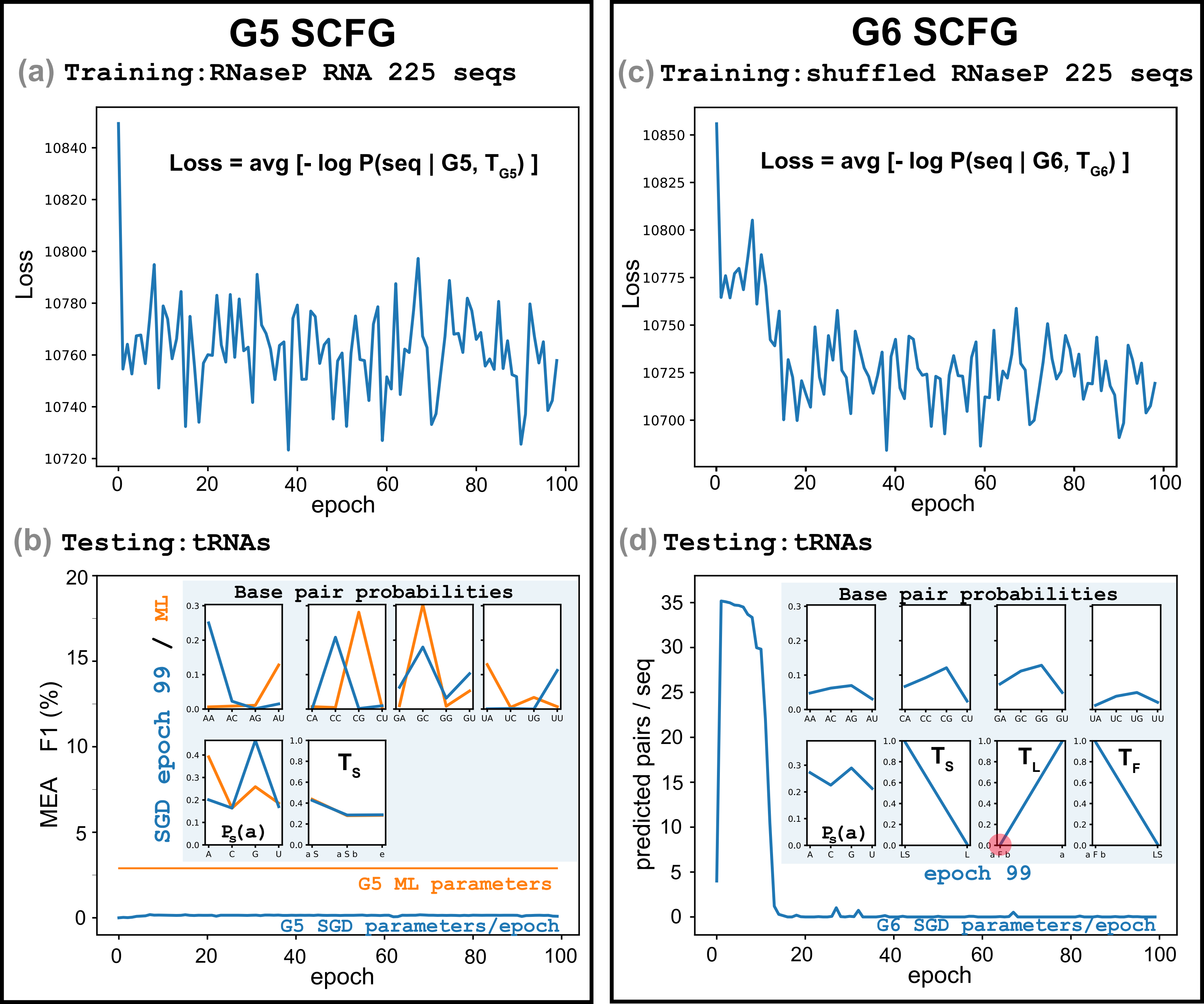

G5 SCFG
(a) Training:RNaseP RNA 225 seqs
Loss = avg [- log P(seq | G5, T_{G5})]
Loss
epoch
(b) Testing:tRNAs
Base pair probabilities
SGD epoch 99 / ML
P_S(a)
T_S
G5 ML parameters
G5 SGD parameters/epoch
MEA F1 (%)
epoch
G6 SCFG
(c) Training:shuffled RNaseP 225 seqs
Loss = avg [- log P(seq | G6, T_{G6})]
Loss
epoch
(d) Testing:tRNAs
Base pair probabilities
P_S(a)
T_S
T_L
T_F
epoch 99
G6 SGD parameters/epoch
predicted pairs / seq
epoch
