# Supplementary figures and images for: What does it take to learn the rules of RNA base pairing? A lot less than you may think
Source: bioRxiv. 2025 Aug 2:2025.07.31.668042. Preprint. [Version 1] doi: 10.1101/2025.07.31.668042 (PMC12324431; doi:10.1101/2025.07.31.668042)

# G6 SCFG

(a) Training: 7 RNA Fams 400 seqs

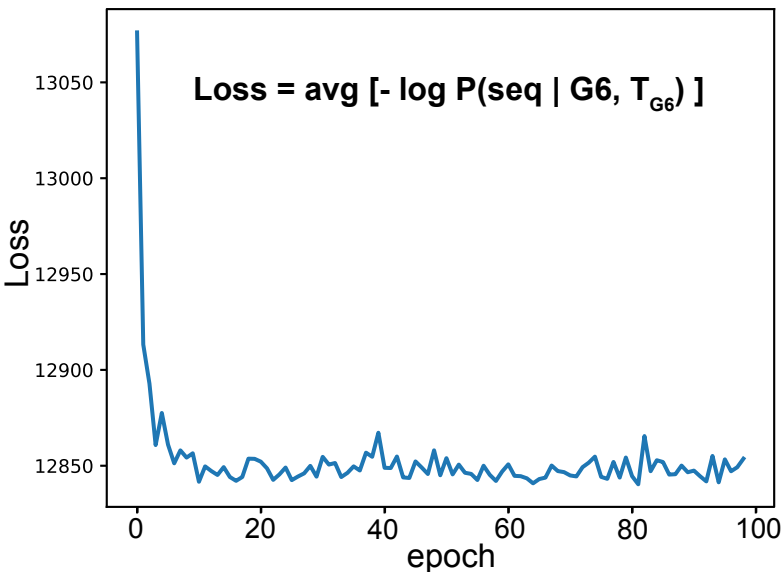

(b) Testing: tRNAs

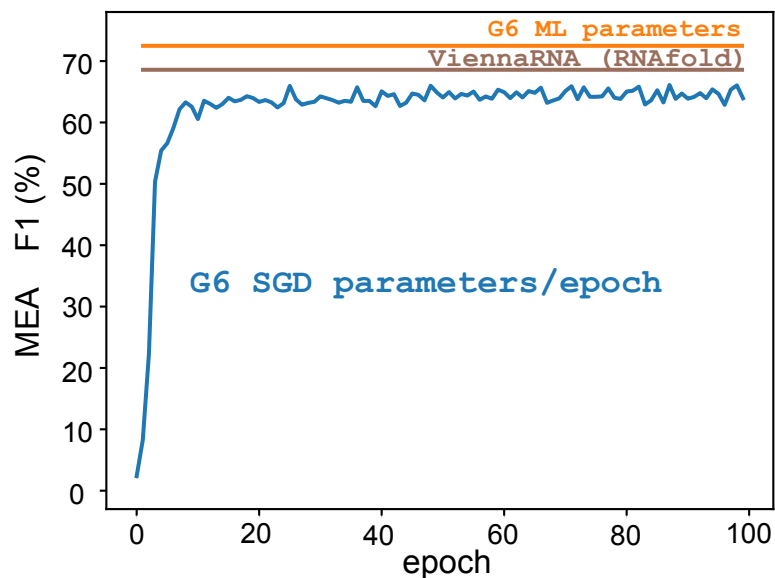

(c) Parameter optimization

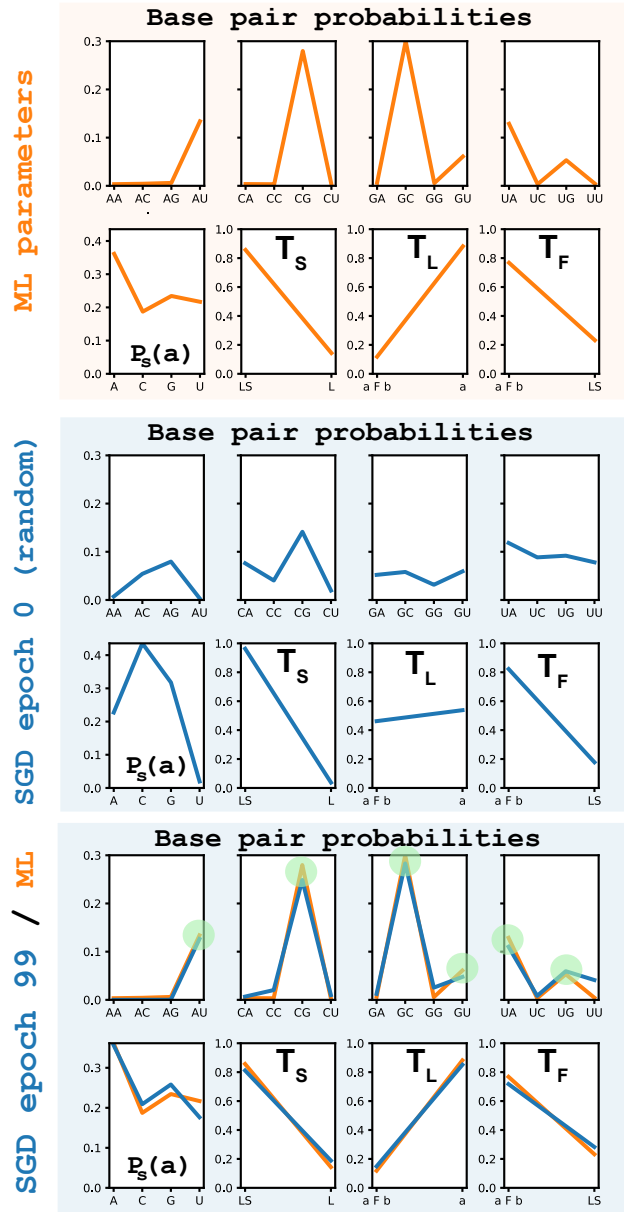

Supplement: Supplement 1 [file media-1.gz › supplemental_material/Figures_data/Figure_3/Figure_R2.pdf]

# G6 SCFG

(a) Training: RNaseP RNA 50 seqs

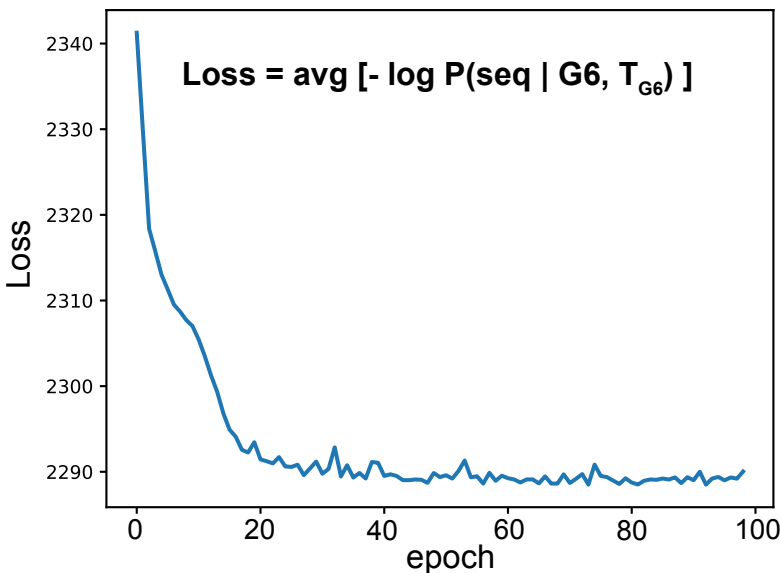

(b) Testing: tRNAs

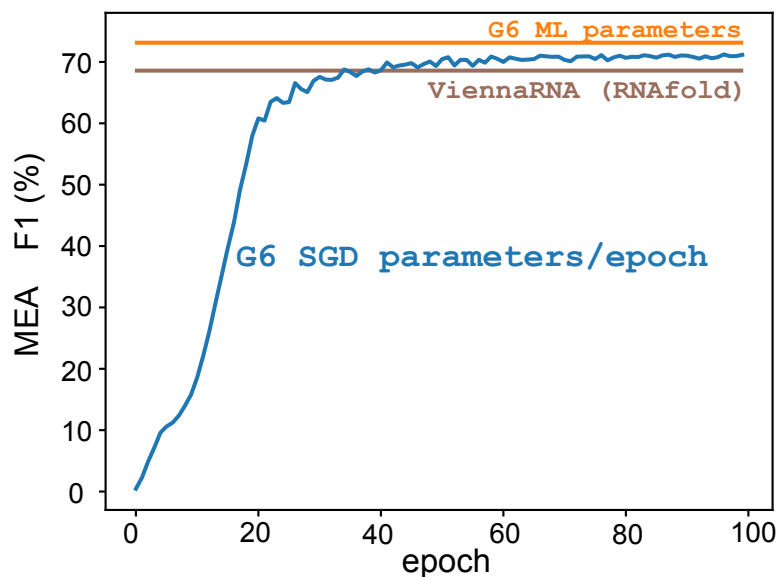

(c) Parameter optimization

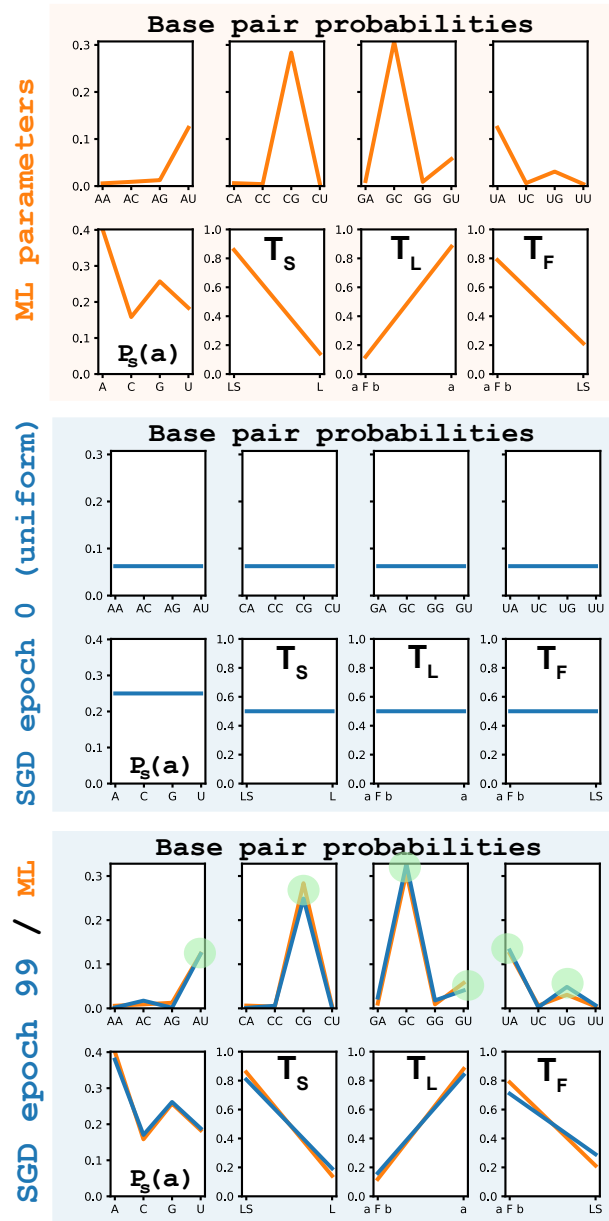

Supplement: Supplement 1 [file media-1.gz › supplemental_material/Figures_data/Figure_2/Figure_R1.pdf]

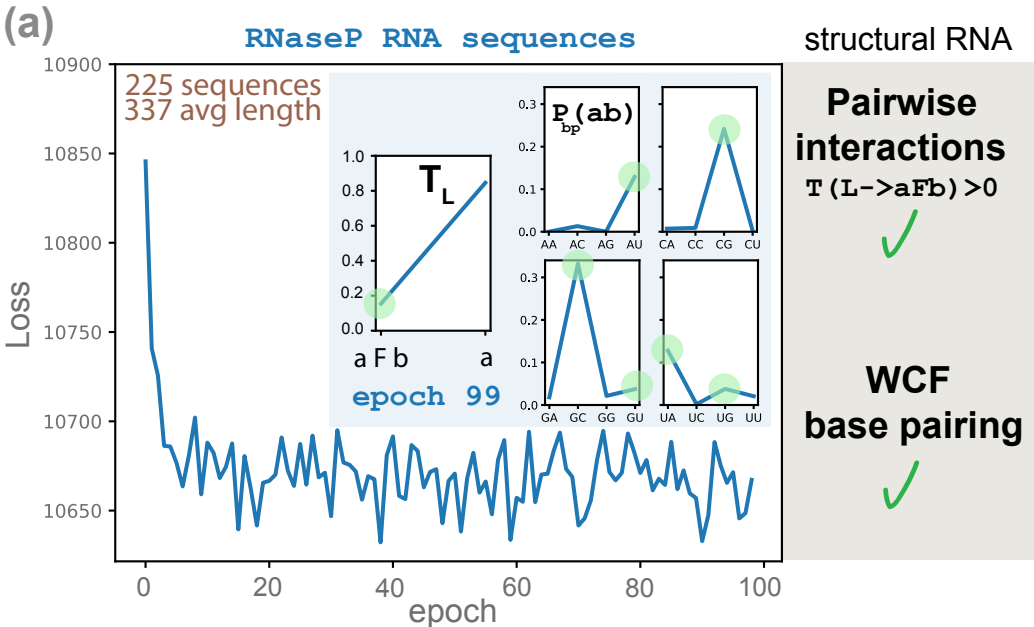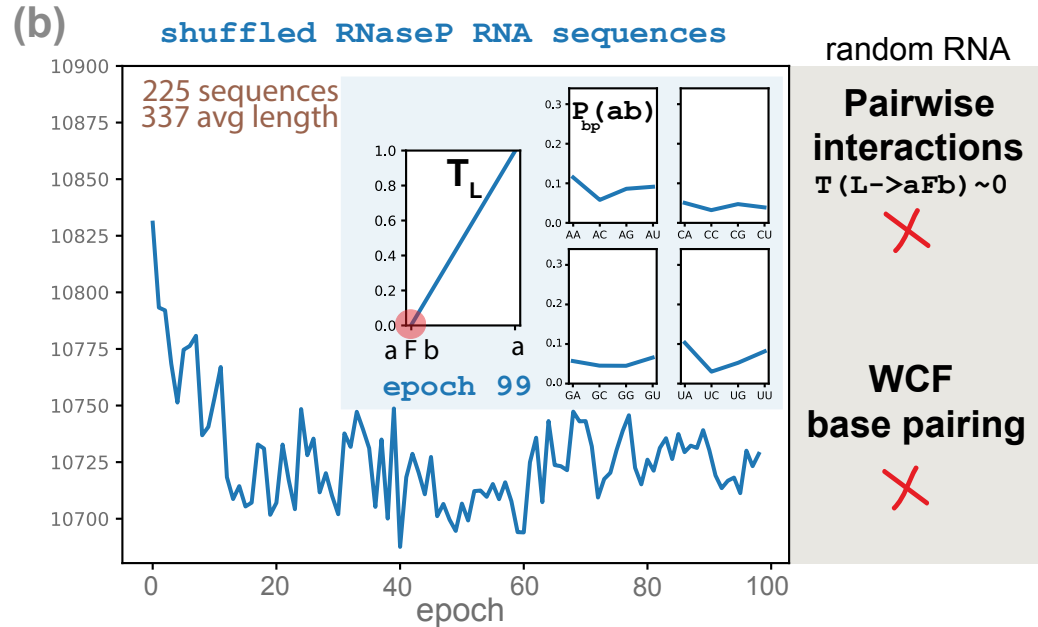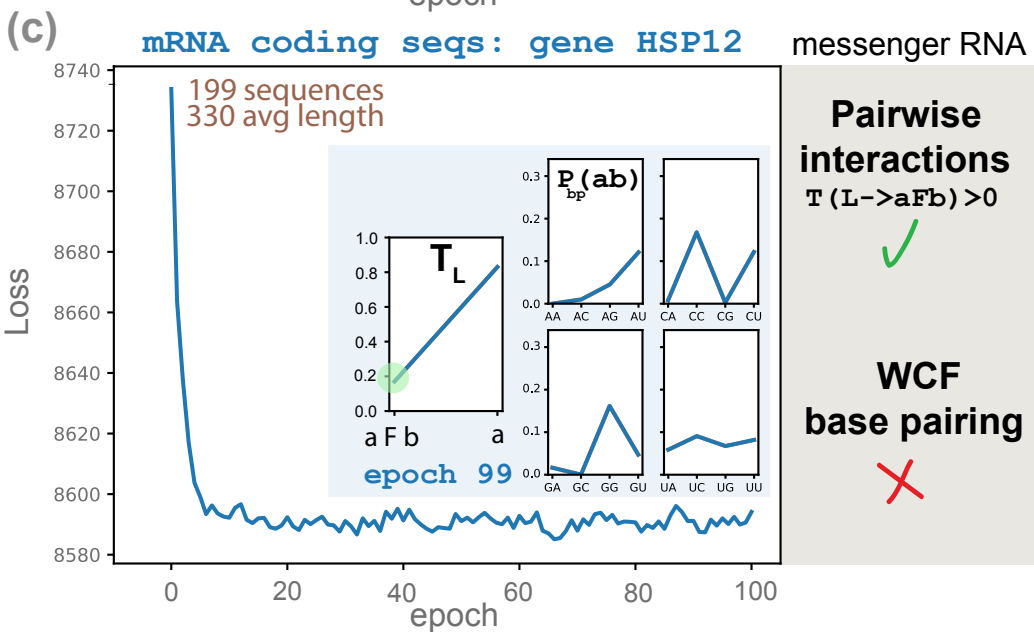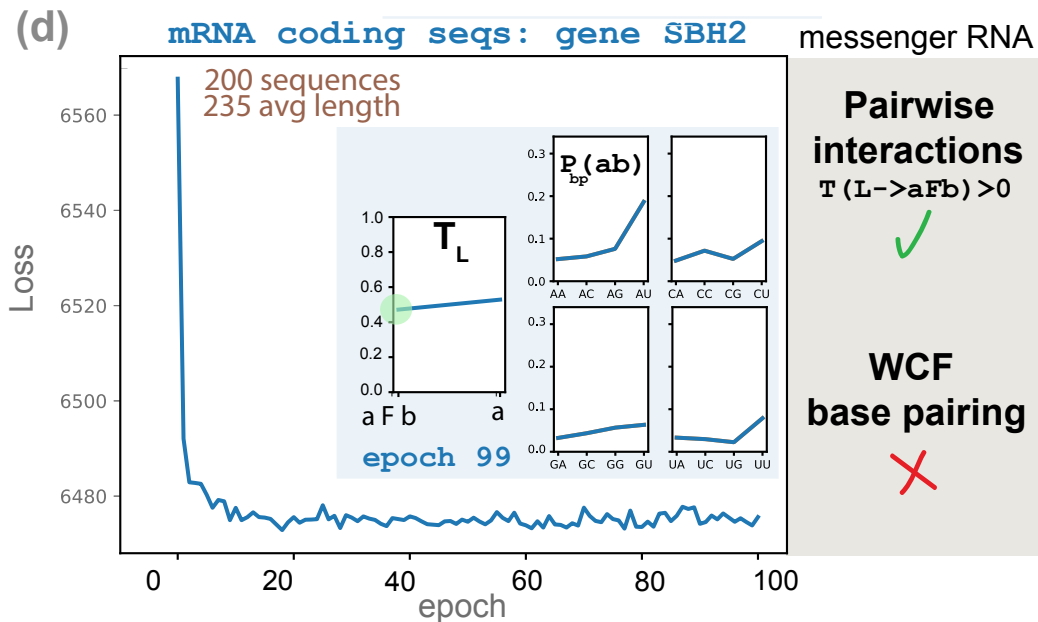

Supplement: Supplement 1 [file media-1.gz › supplemental_material/Figures_data/Figure_5/Figure_R4.pdf]

# G6 SCFG

(a) Training: RNaseP RNA 25 seqs

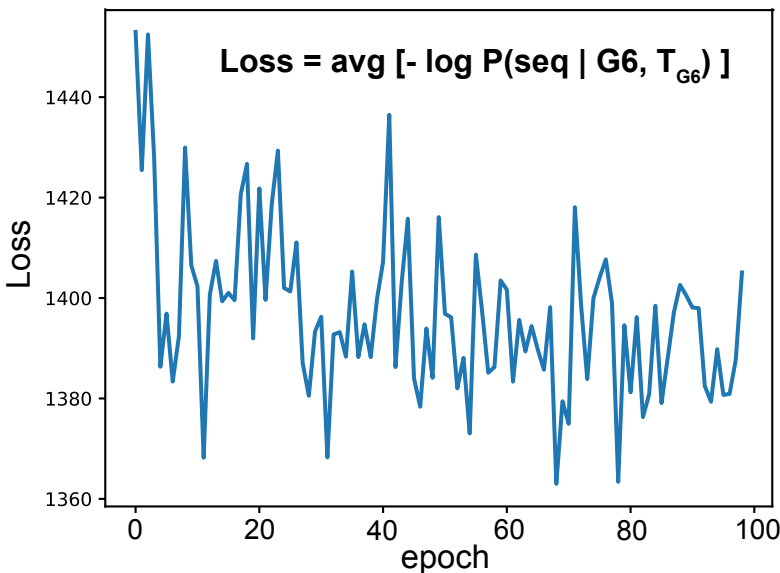

(b) Testing: tRNAs

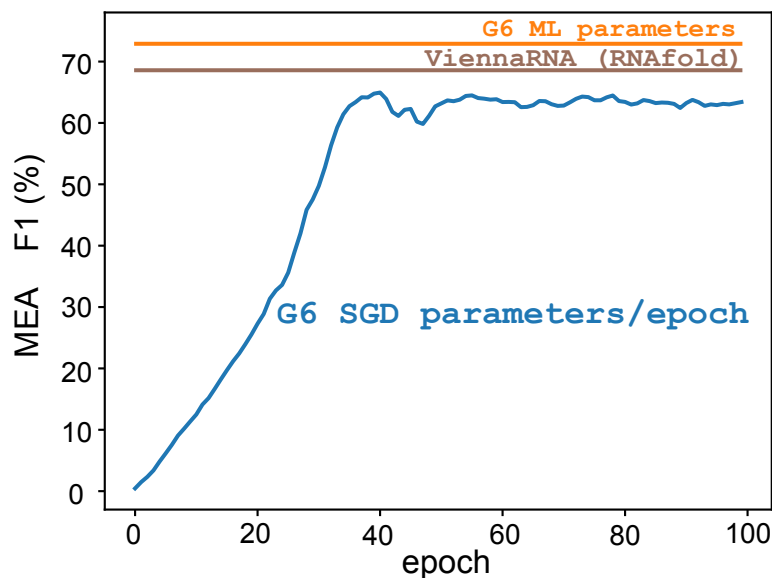

(c) Parameter optimization

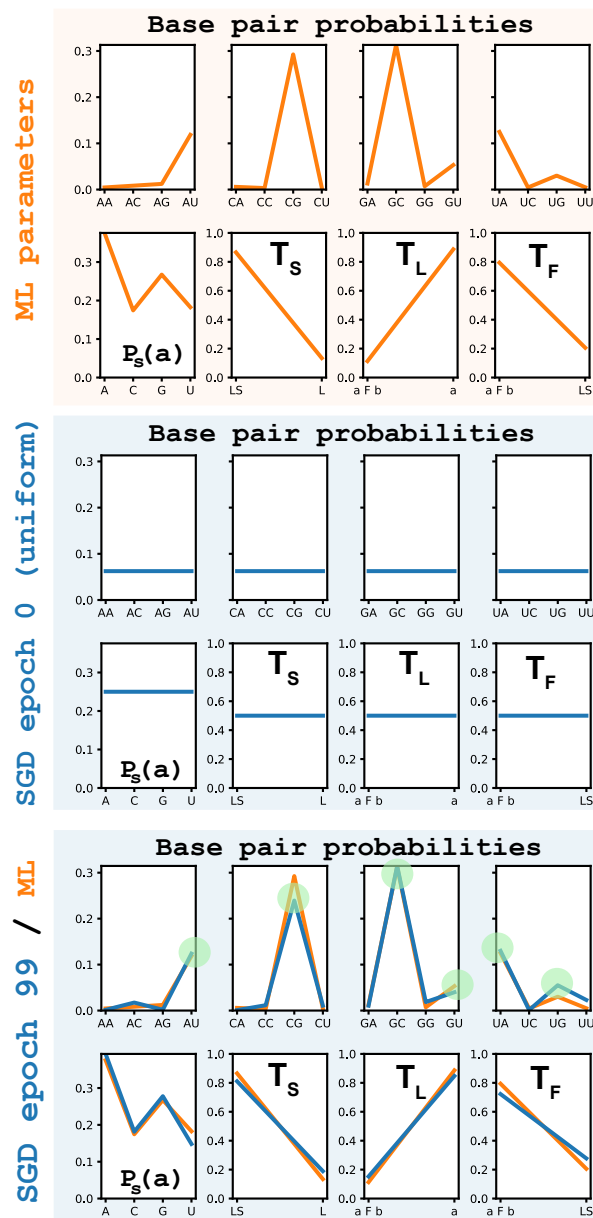

Supplement: Supplement 1 [file media-1.gz › supplemental_material/Figures_data/Figure_S1/Figure_S1.pdf]

# G6 grammar TORNADO\_conus\_rnabench\_RNaseP\_g6

Pair Probabilities  $P(ab)$  [ $\sum_{ab} P(ab) = 1$ ]  $a, b = \{A, C, G, U\}$

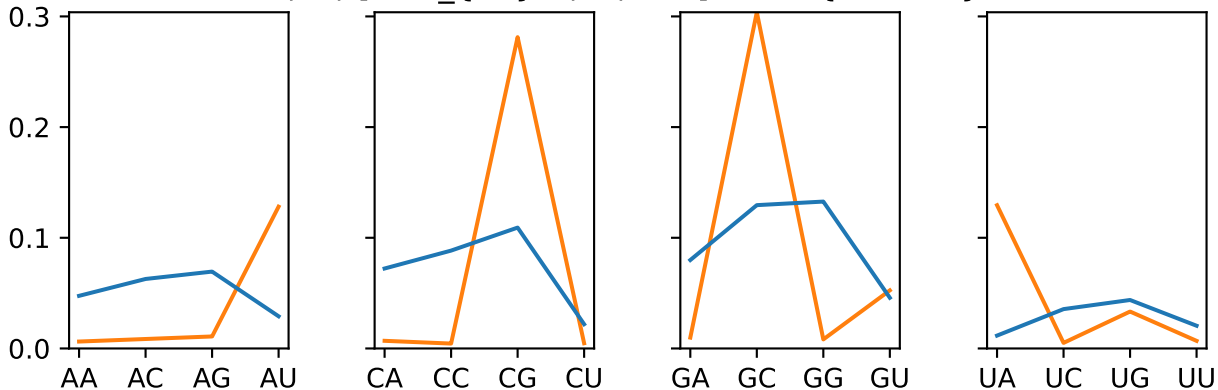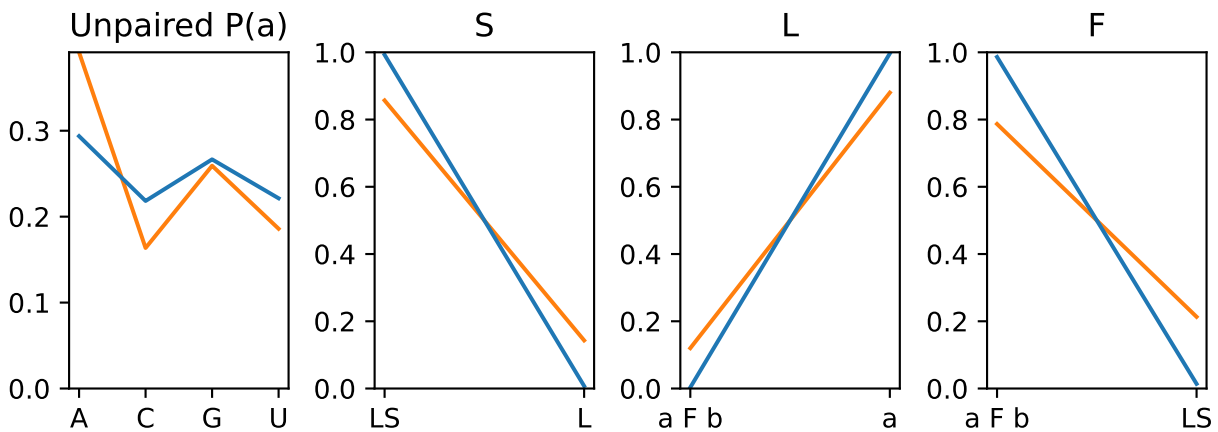

Supplement: Supplement 1 [file media-1.gz › supplemental_material/experiments/g6_optimize_param_conus_rnabench_RNaseP_uniform_shuffle/g6_params_i62.pdf]

# G6 grammar TORNADO\_conus\_rnabench\_RNaseP\_g6

Pair Probabilities  $P(ab)$  [ $\sum_{ab} P(ab) = 1$ ]  $a, b = \{A, C, G, U\}$

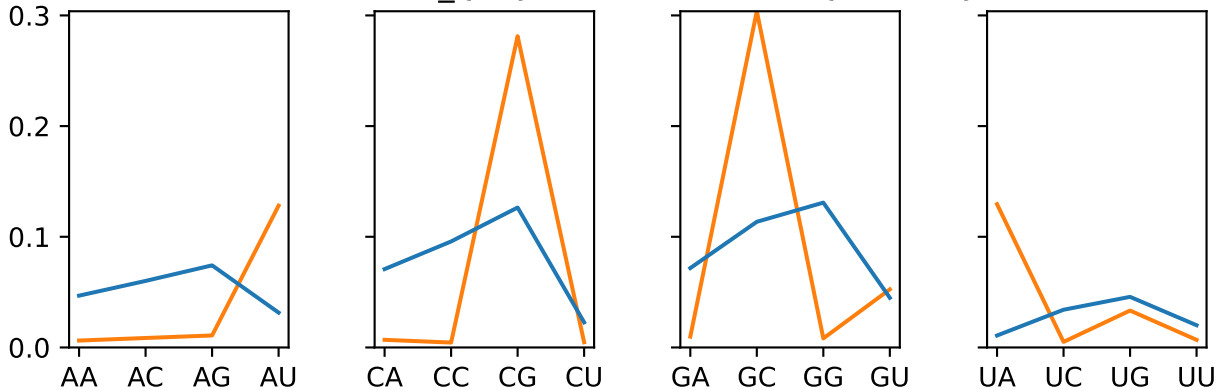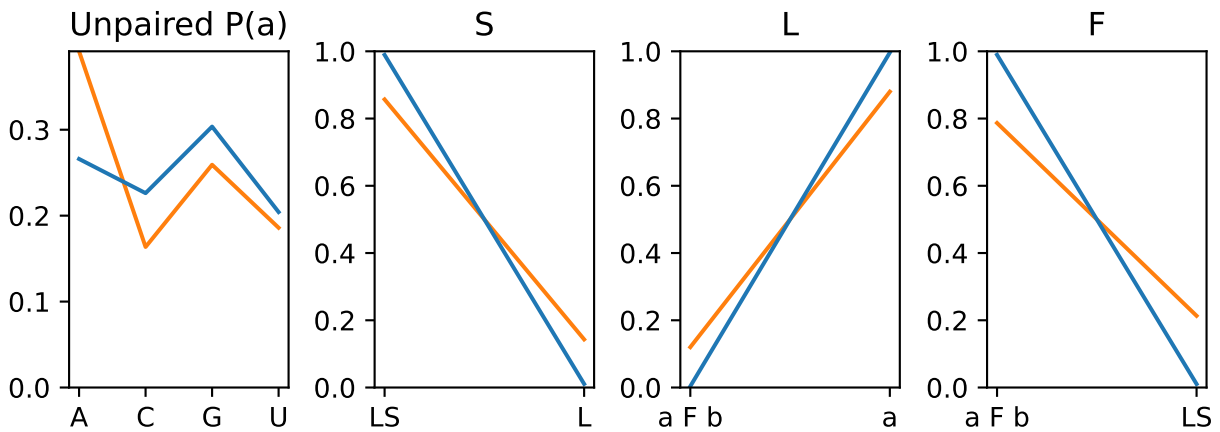

Supplement: Supplement 1 [file media-1.gz › supplemental_material/experiments/g6_optimize_param_conus_rnabench_RNaseP_uniform_shuffle/g6_params_i76.pdf]

# G6 grammar TORNADO\_conus\_rnabench\_RNaseP\_g6

Pair Probabilities  $P(ab)$  [ $\sum_{ab} P(ab) = 1$ ]  $a, b = \{A, C, G, U\}$

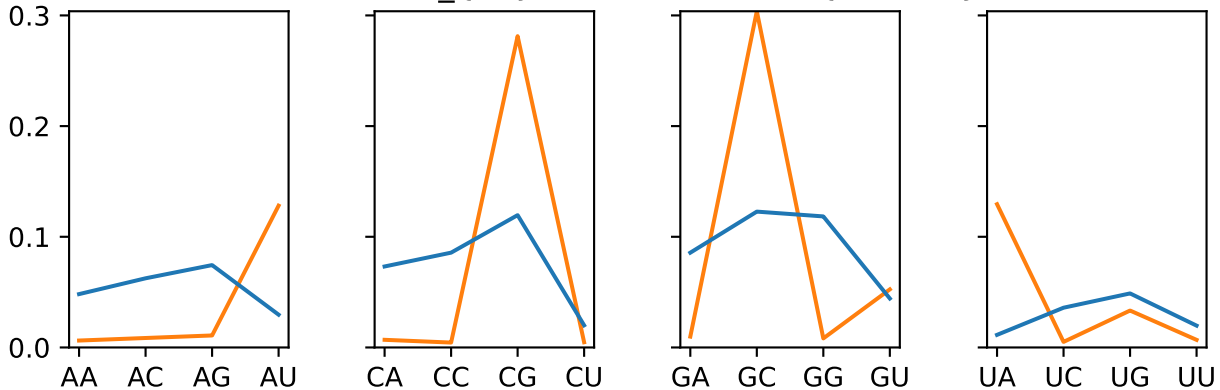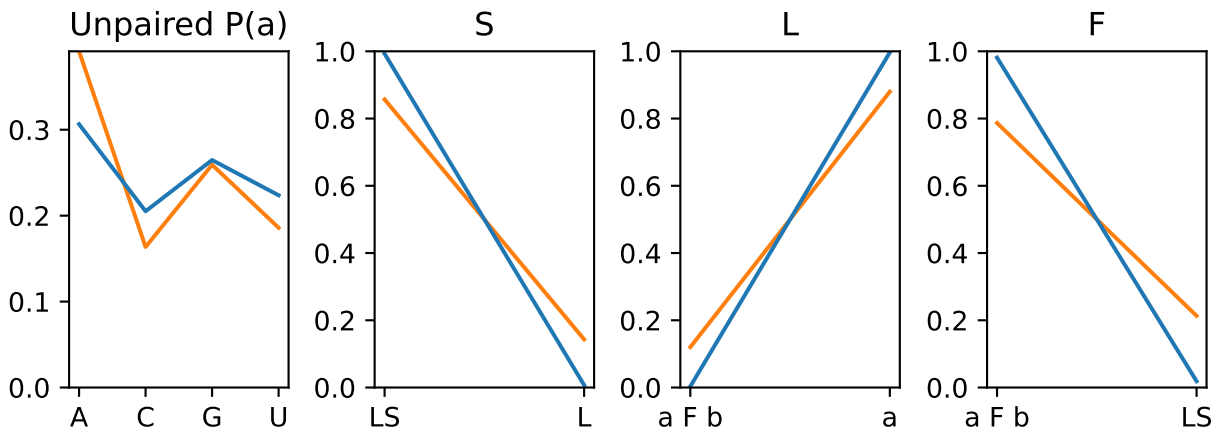

Supplement: Supplement 1 [file media-1.gz › supplemental_material/experiments/g6_optimize_param_conus_rnabench_RNaseP_uniform_shuffle/g6_params_i89.pdf]

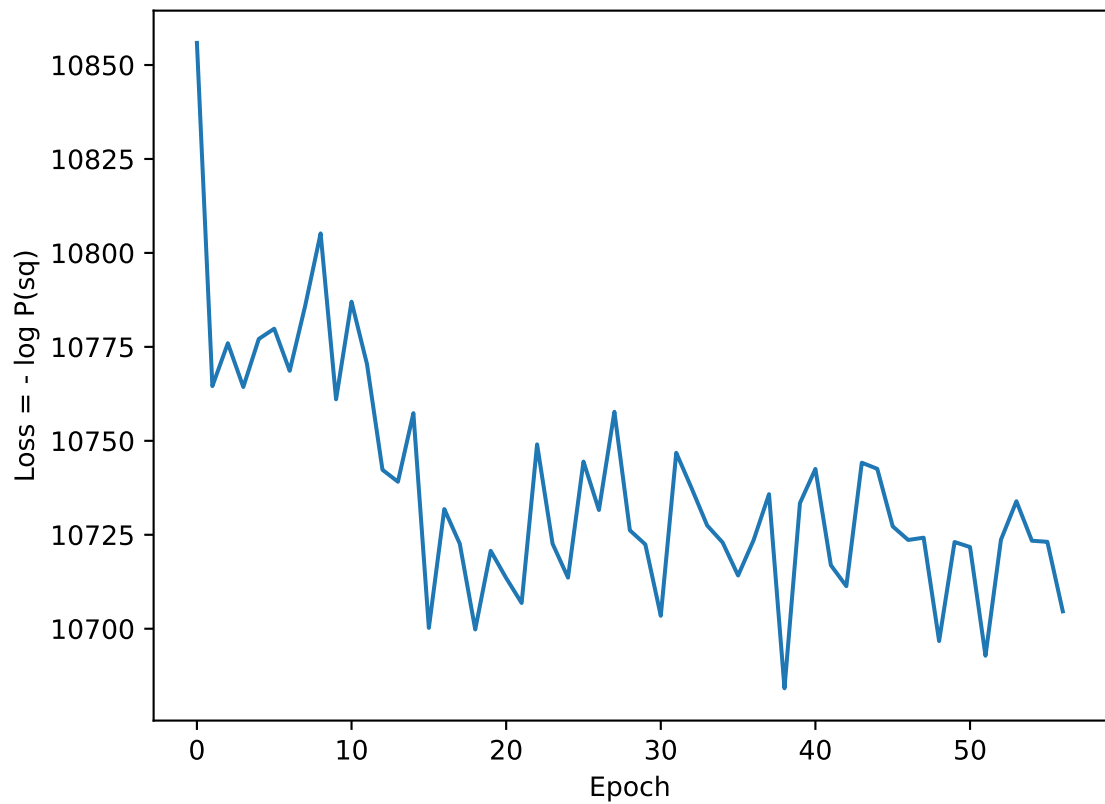

Supplement: Supplement 1 [file media-1.gz › supplemental_material/experiments/g6_optimize_param_conus_rnabench_RNaseP_uniform_shuffle/losses_i57.pdf]

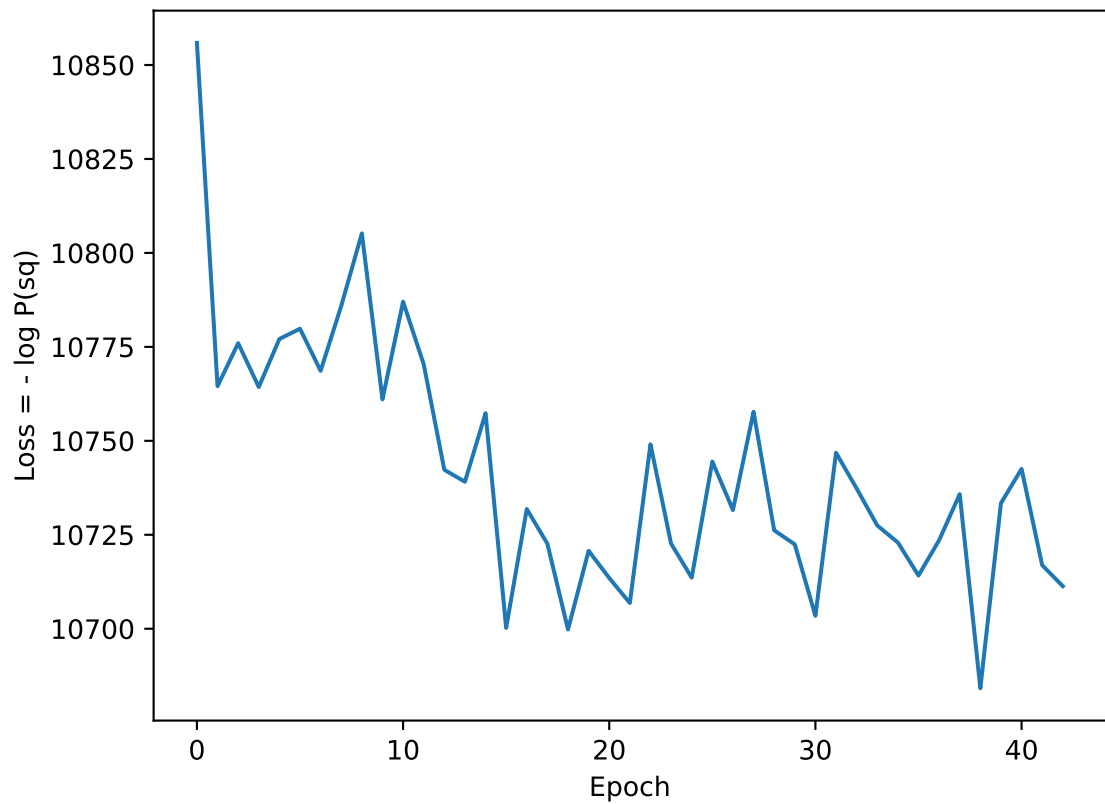

Supplement: Supplement 1 [file media-1.gz › supplemental_material/experiments/g6_optimize_param_conus_rnabench_RNaseP_uniform_shuffle/losses_i43.pdf]

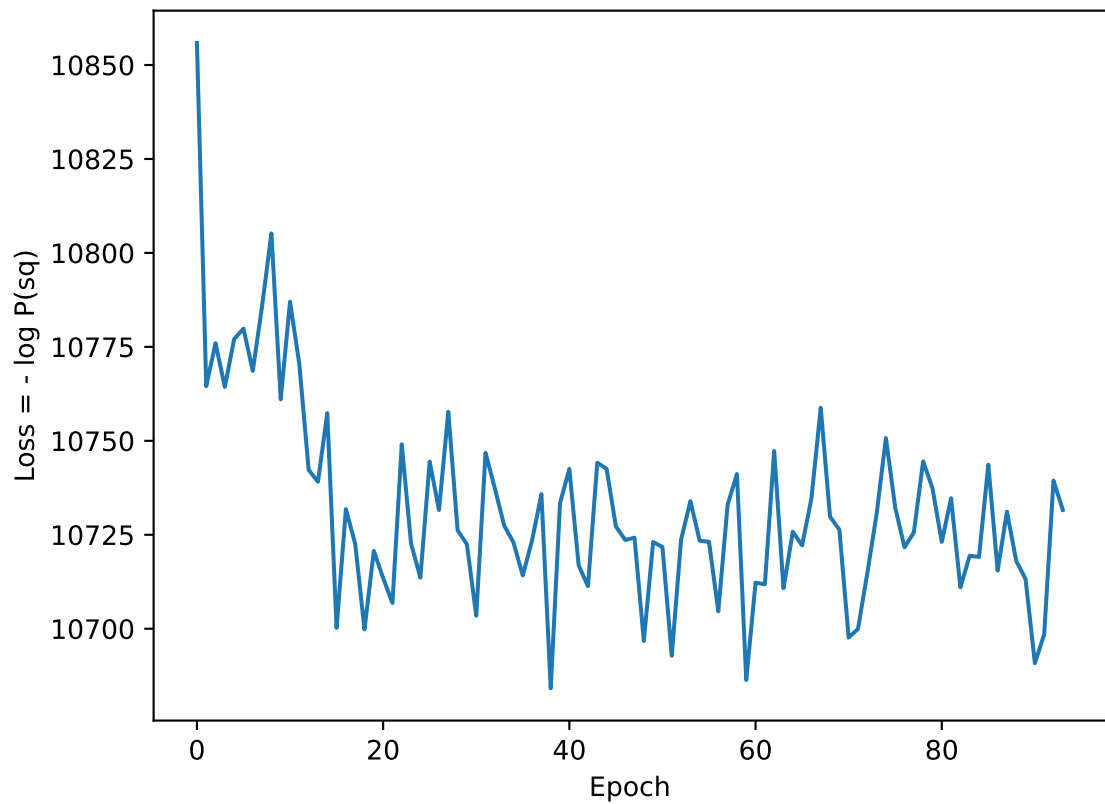

Supplement: Supplement 1 [file media-1.gz › supplemental_material/experiments/g6_optimize_param_conus_rnabench_RNaseP_uniform_shuffle/losses_i94.pdf]

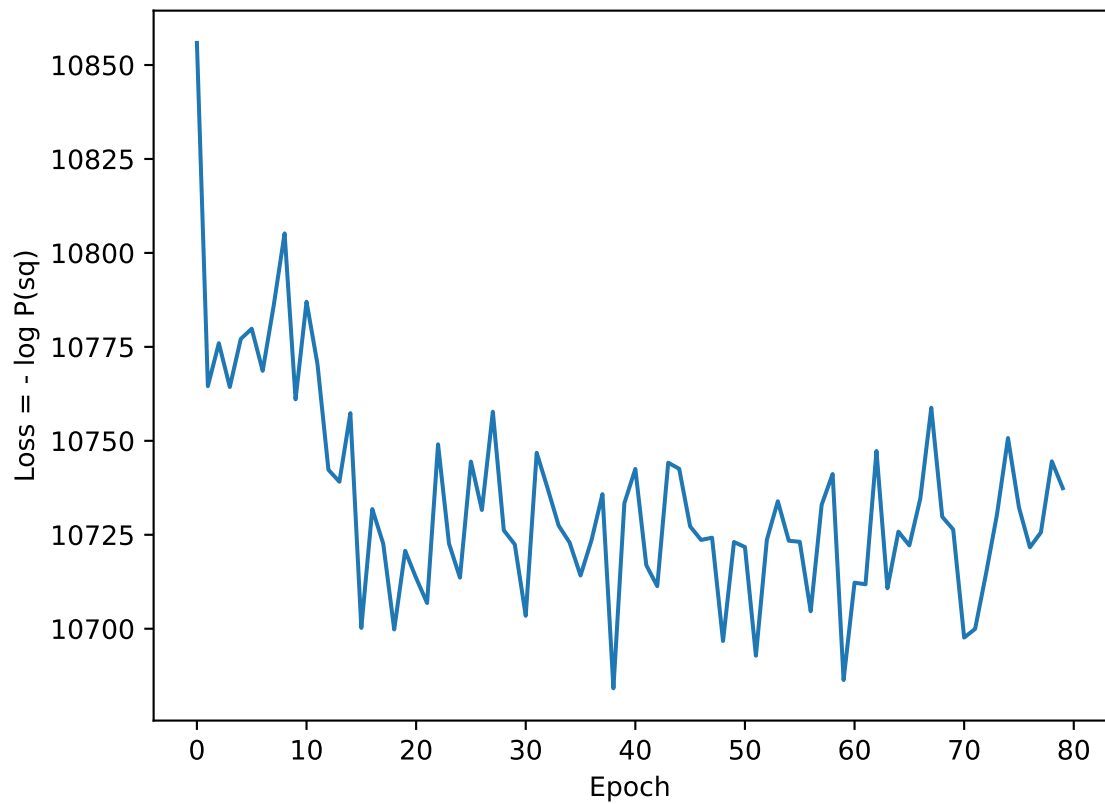

Supplement: Supplement 1 [file media-1.gz › supplemental_material/experiments/g6_optimize_param_conus_rnabench_RNaseP_uniform_shuffle/losses_i80.pdf]

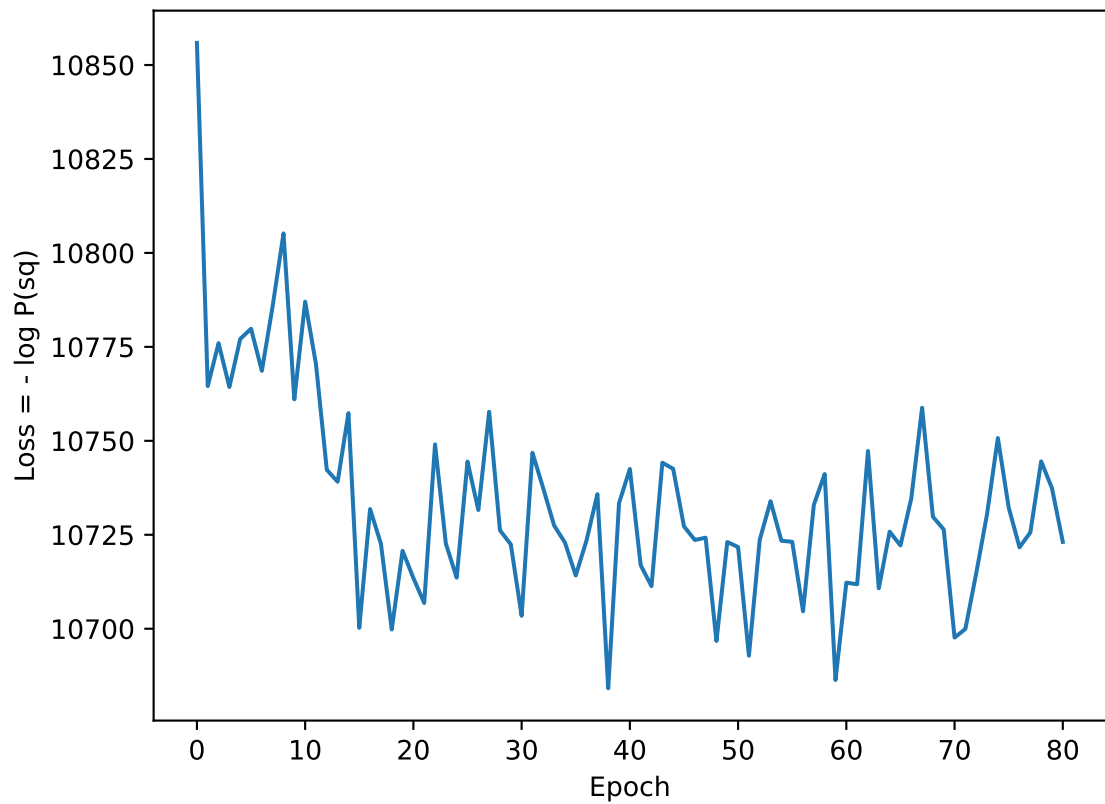

Supplement: Supplement 1 [file media-1.gz › supplemental_material/experiments/g6_optimize_param_conus_rnabench_RNaseP_uniform_shuffle/losses_i81.pdf]

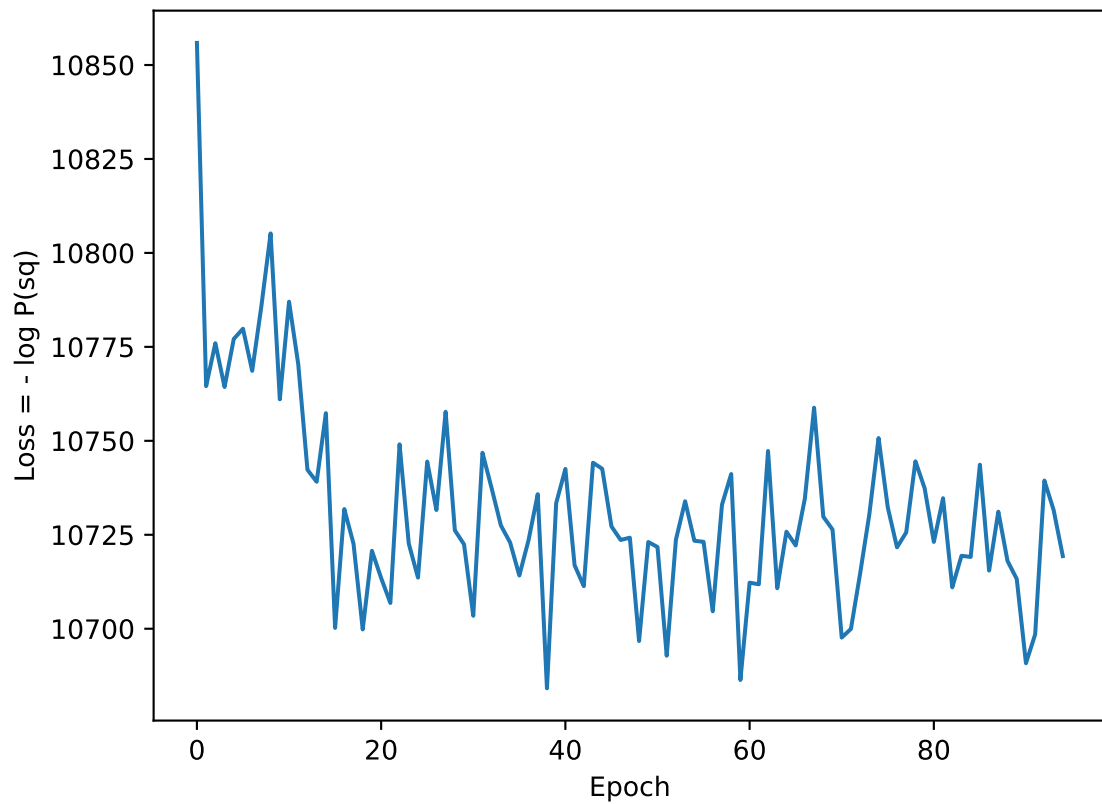

Supplement: Supplement 1 [file media-1.gz › supplemental_material/experiments/g6_optimize_param_conus_rnabench_RNaseP_uniform_shuffle/losses_i95.pdf]

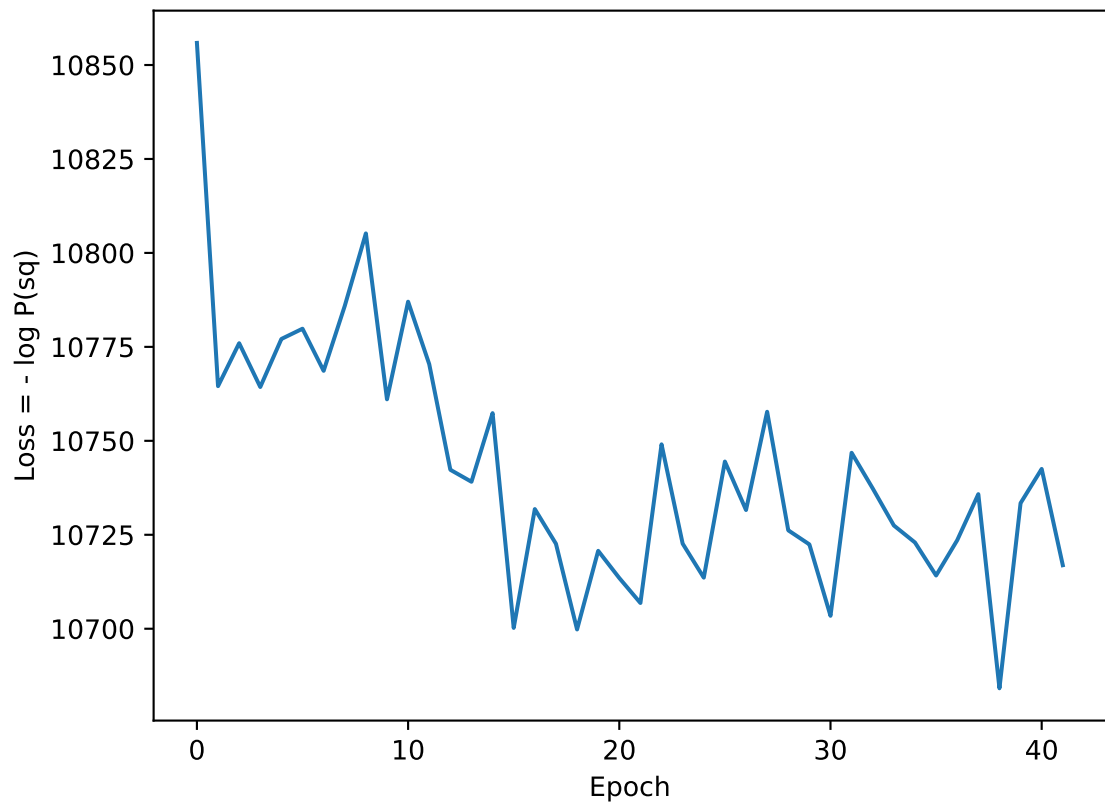

Supplement: Supplement 1 [file media-1.gz › supplemental_material/experiments/g6_optimize_param_conus_rnabench_RNaseP_uniform_shuffle/losses_i42.pdf]

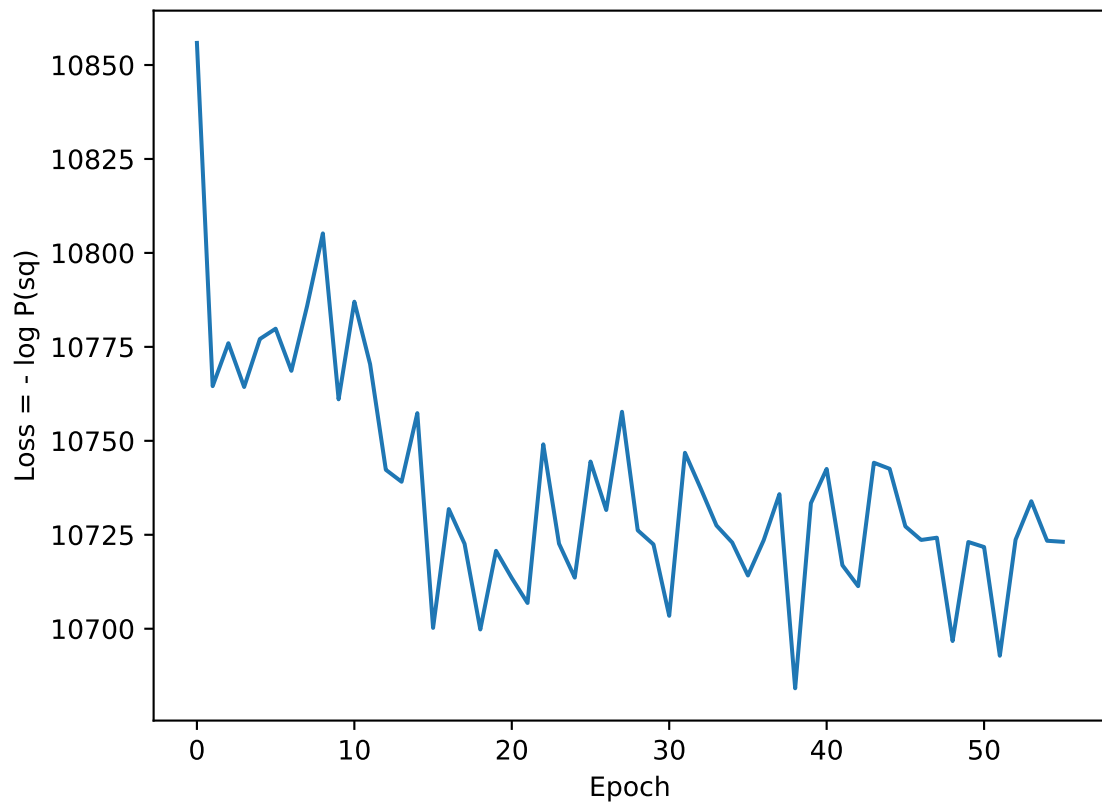

Supplement: Supplement 1 [file media-1.gz › supplemental_material/experiments/g6_optimize_param_conus_rnabench_RNaseP_uniform_shuffle/losses_i56.pdf]

# G6 grammar TORNADO\_conus\_rnabench\_RNaseP\_g6

Pair Probabilities  $P(ab)$  [ $\sum_{ab} P(ab) = 1$ ]  $a, b = \{A, C, G, U\}$

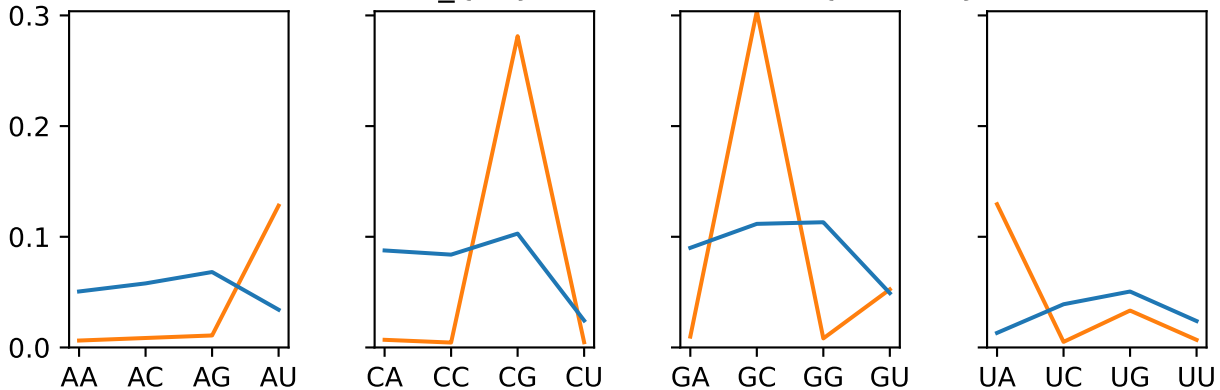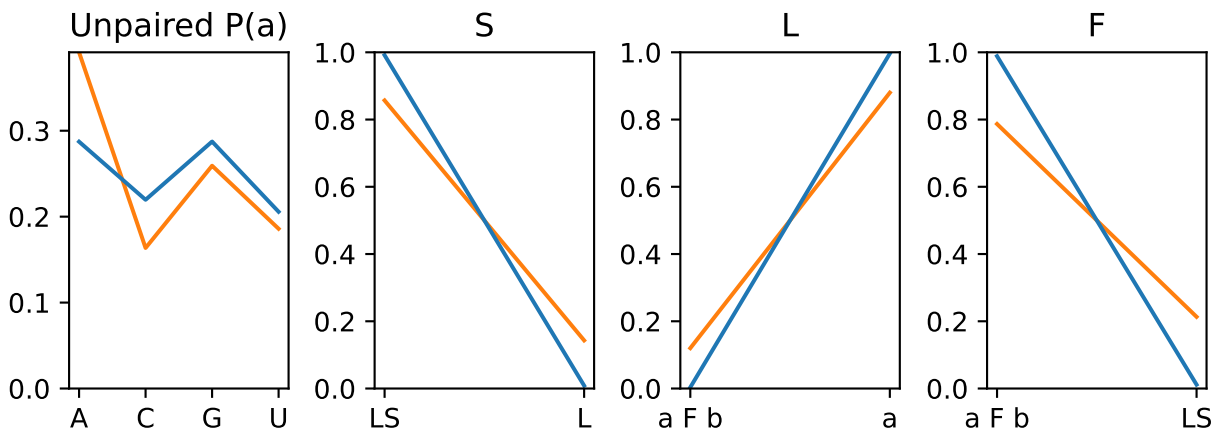

Supplement: Supplement 1 [file media-1.gz › supplemental_material/experiments/g6_optimize_param_conus_rnabench_RNaseP_uniform_shuffle/g6_params_i88.pdf]

# G6 grammar TORNADO\_conus\_rnabench\_RNaseP\_g6

Pair Probabilities  $P(ab)$  [ $\sum_{ab} P(ab) = 1$ ]  $a, b = \{A, C, G, U\}$

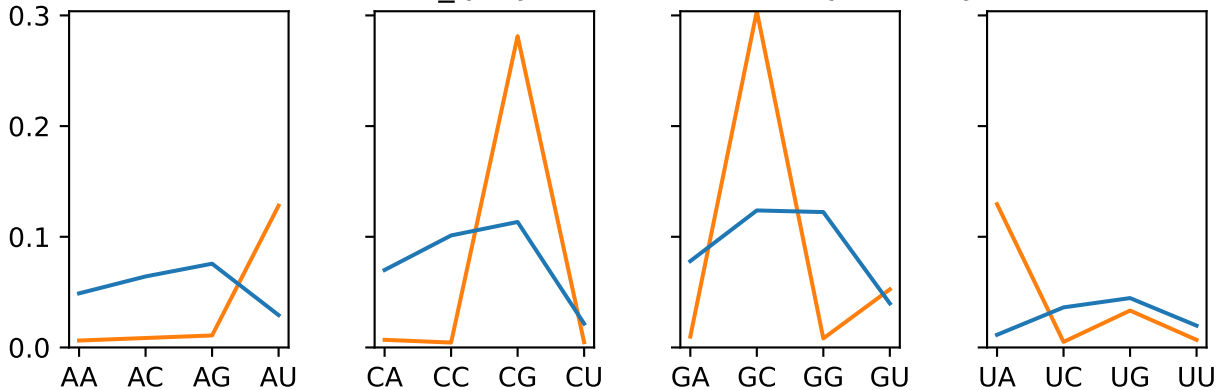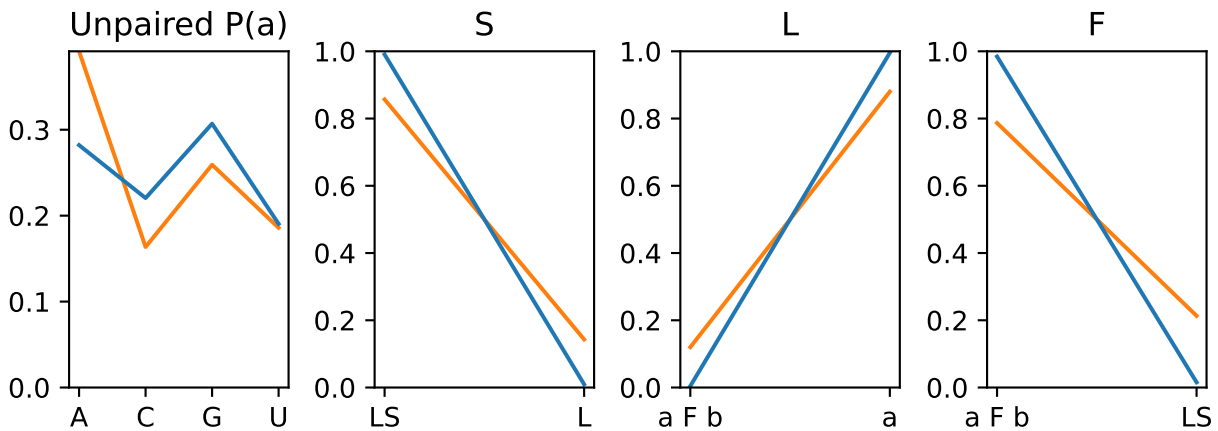

Supplement: Supplement 1 [file media-1.gz › supplemental_material/experiments/g6_optimize_param_conus_rnabench_RNaseP_uniform_shuffle/g6_params_i77.pdf]

# G6 grammar TORNADO\_conus\_rnabench\_RNaseP\_g6

Pair Probabilities  $P(ab)$  [ $\sum_{ab} P(ab) = 1$ ]  $a, b = \{A, C, G, U\}$

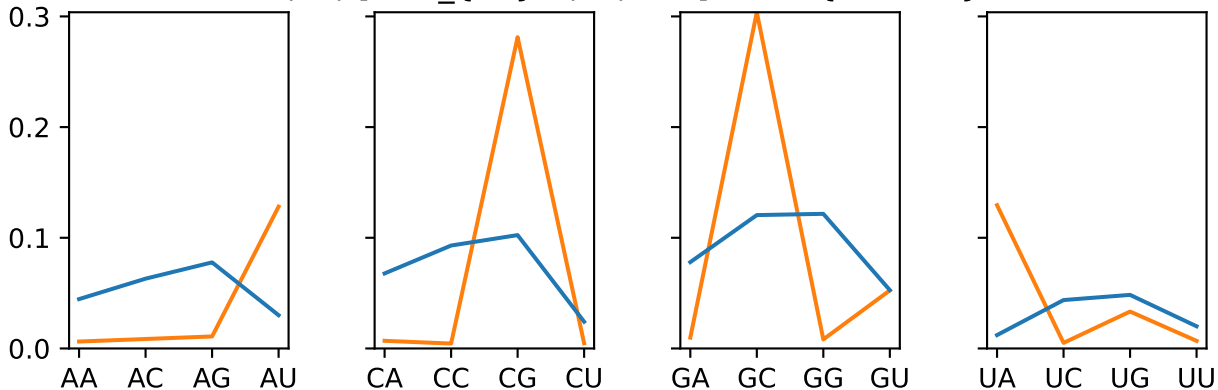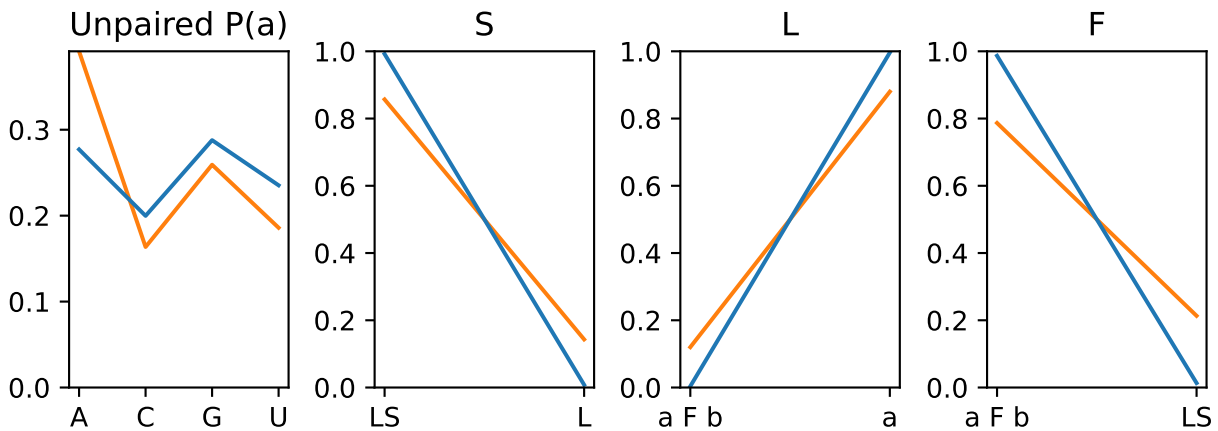

Supplement: Supplement 1 [file media-1.gz › supplemental_material/experiments/g6_optimize_param_conus_rnabench_RNaseP_uniform_shuffle/g6_params_i63.pdf]

# G6 grammar TORNADO\_conus\_rnabench\_RNaseP\_g6

Pair Probabilities  $P(ab)$  [ $\sum_{ab} P(ab) = 1$ ]  $a, b = \{A, C, G, U\}$

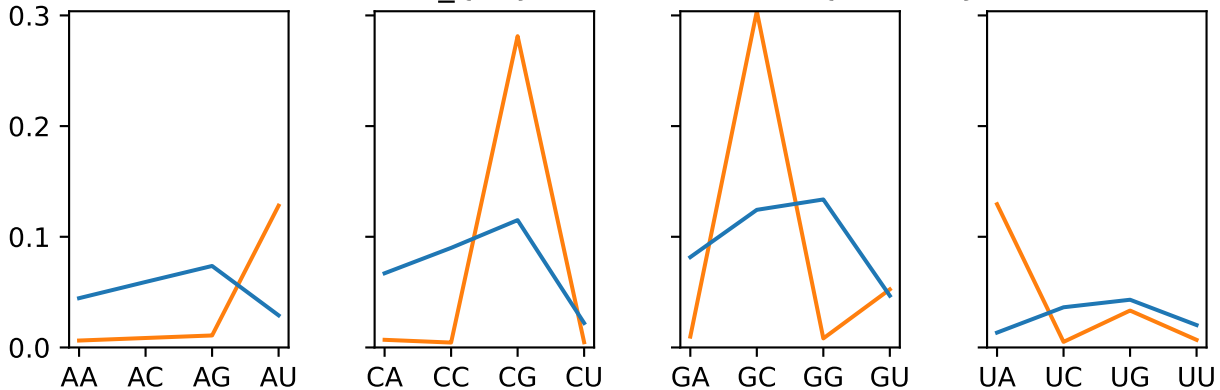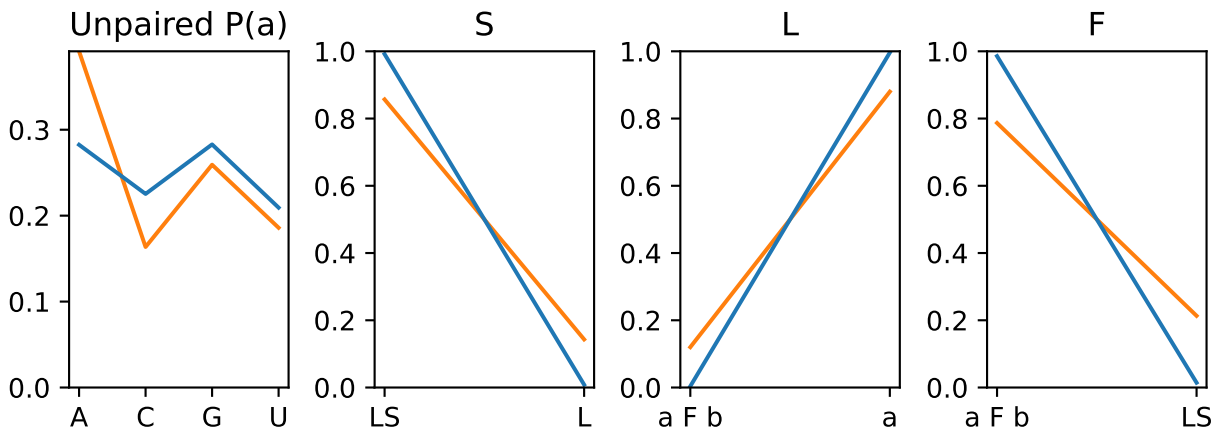

Supplement: Supplement 1 [file media-1.gz › supplemental_material/experiments/g6_optimize_param_conus_rnabench_RNaseP_uniform_shuffle/g6_params_i49.pdf]

# G6 grammar TORNADO\_conus\_rnabench\_RNaseP\_g6

Pair Probabilities  $P(ab)$  [ $\sum_{ab} P(ab) = 1$ ]  $a, b = \{A, C, G, U\}$

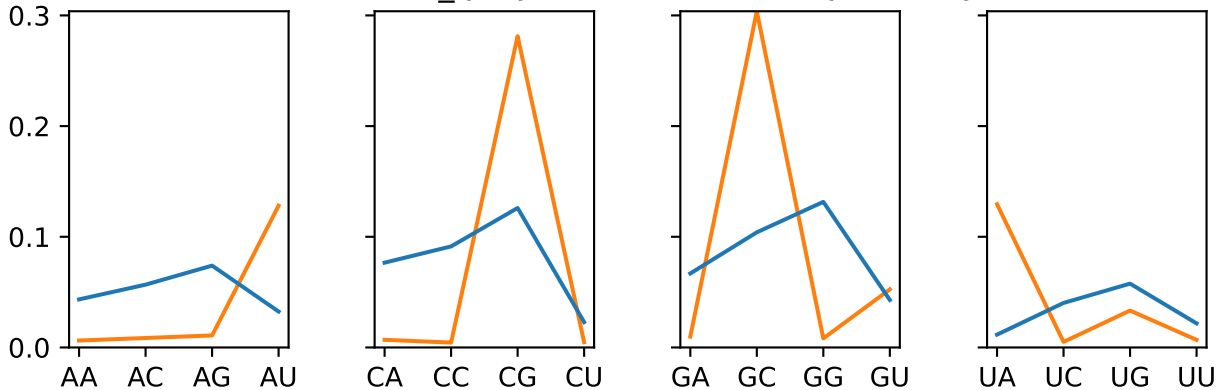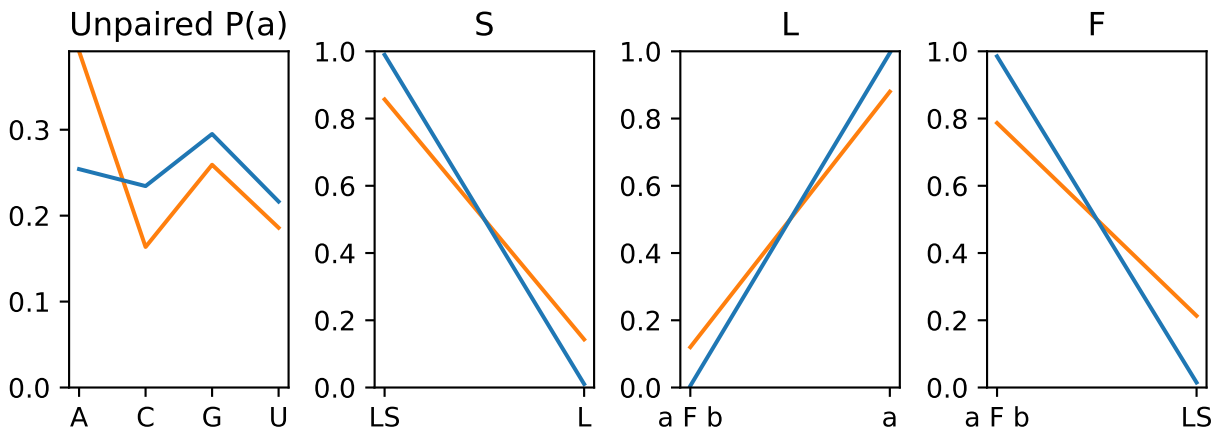

Supplement: Supplement 1 [file media-1.gz › supplemental_material/experiments/g6_optimize_param_conus_rnabench_RNaseP_uniform_shuffle/g6_params_i75.pdf]

# G6 grammar TORNADO\_conus\_rnabench\_RNaseP\_g6

Pair Probabilities  $P(ab)$  [ $\sum_{ab} P(ab) = 1$ ]  $a, b = \{A, C, G, U\}$

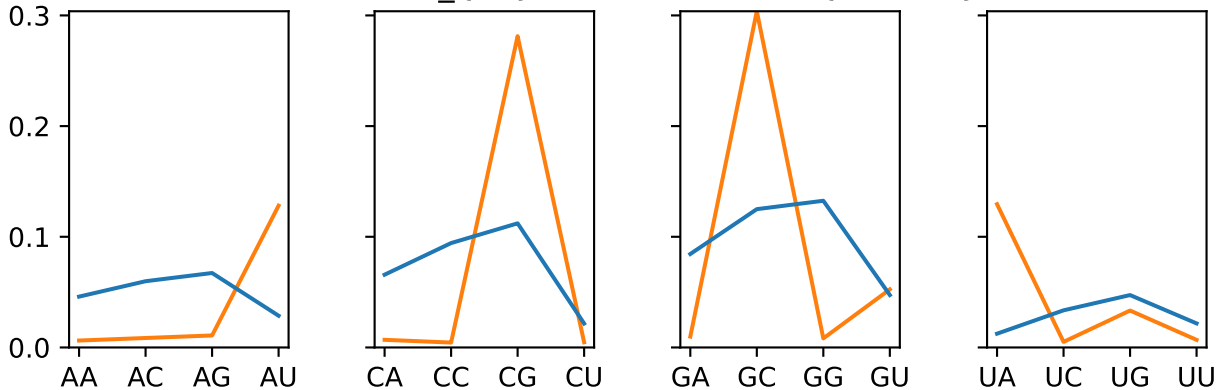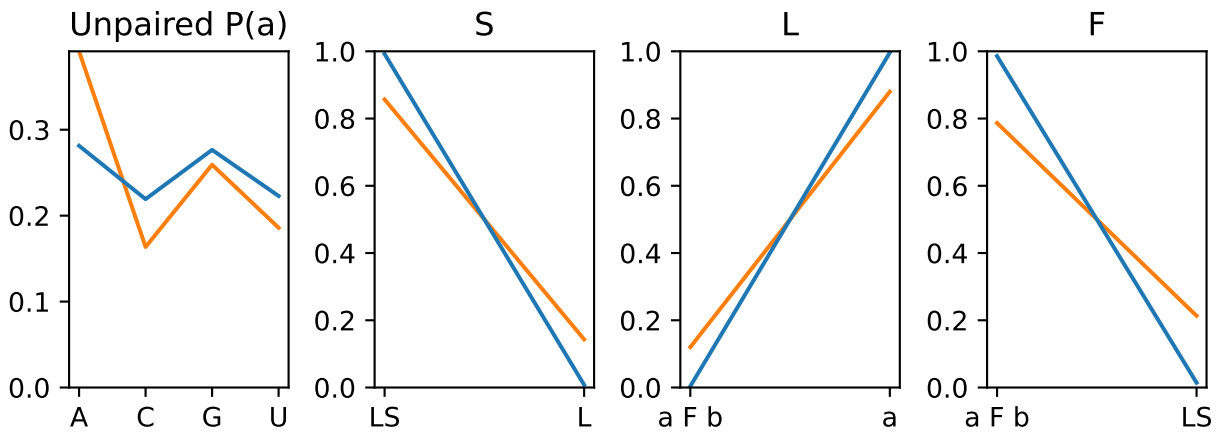

Supplement: Supplement 1 [file media-1.gz › supplemental_material/experiments/g6_optimize_param_conus_rnabench_RNaseP_uniform_shuffle/g6_params_i61.pdf]

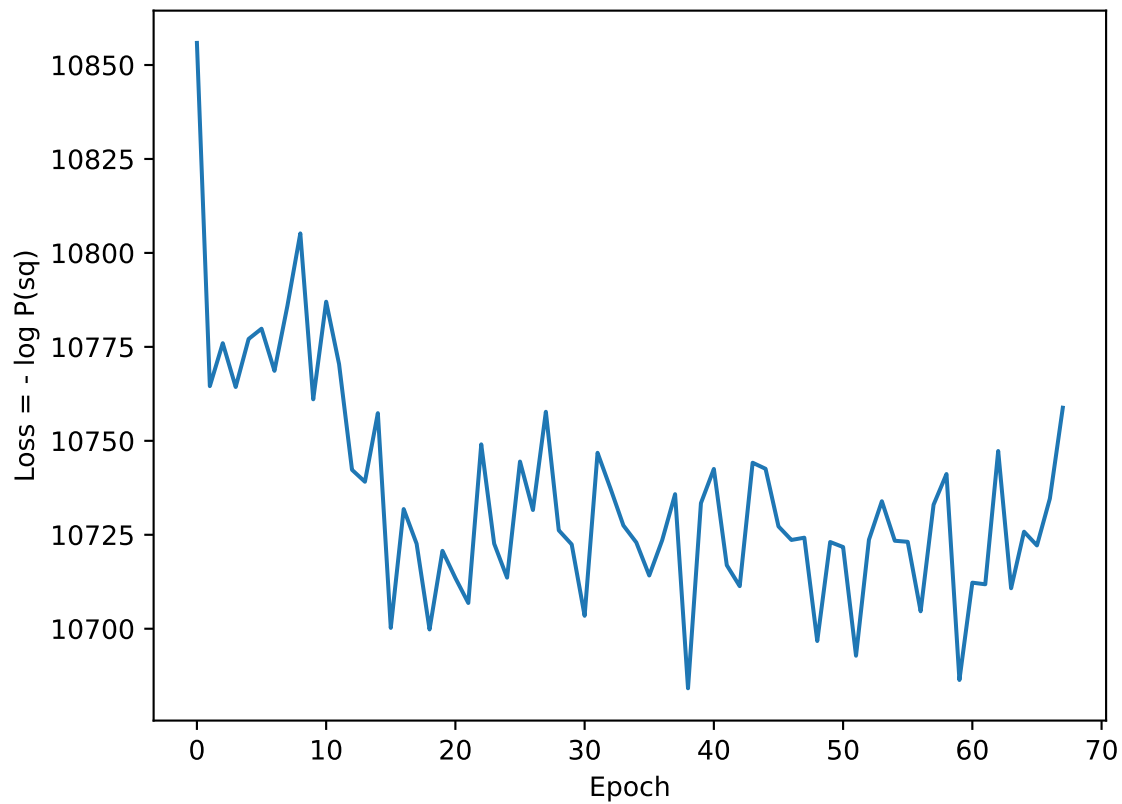

Supplement: Supplement 1 [file media-1.gz › supplemental_material/experiments/g6_optimize_param_conus_rnabench_RNaseP_uniform_shuffle/losses_i68.pdf]

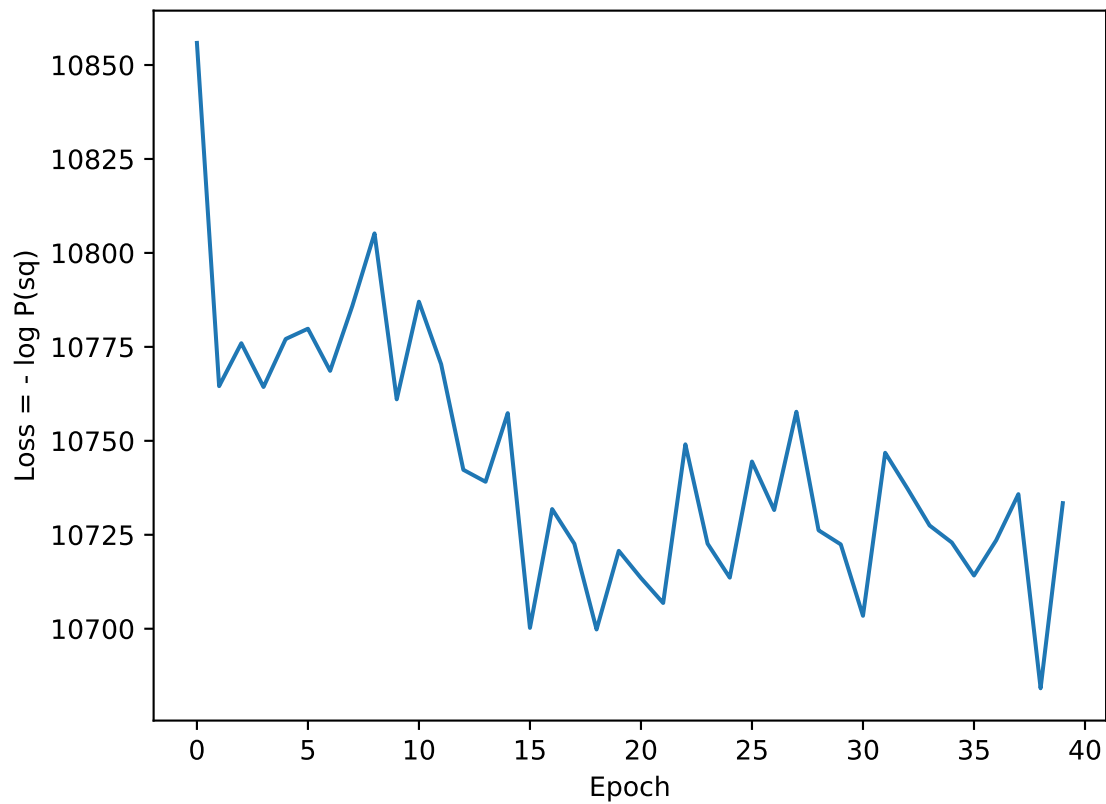

Supplement: Supplement 1 [file media-1.gz › supplemental_material/experiments/g6_optimize_param_conus_rnabench_RNaseP_uniform_shuffle/losses_i40.pdf]

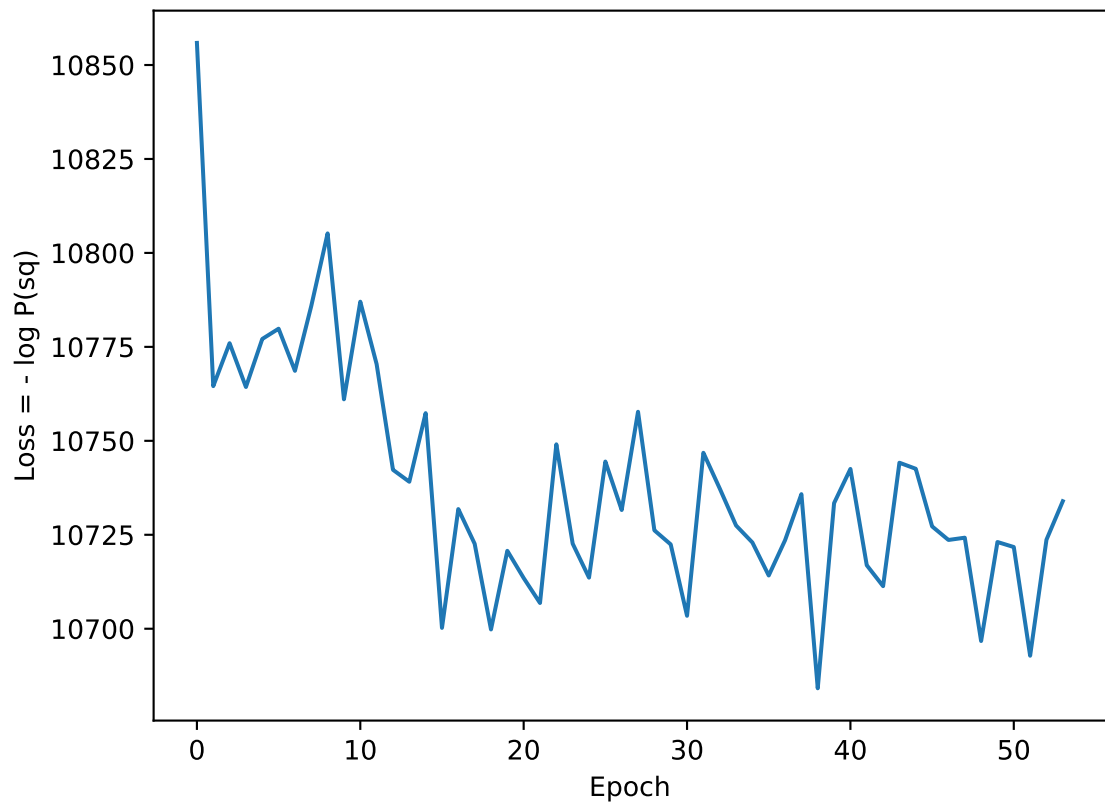

Supplement: Supplement 1 [file media-1.gz › supplemental_material/experiments/g6_optimize_param_conus_rnabench_RNaseP_uniform_shuffle/losses_i54.pdf]

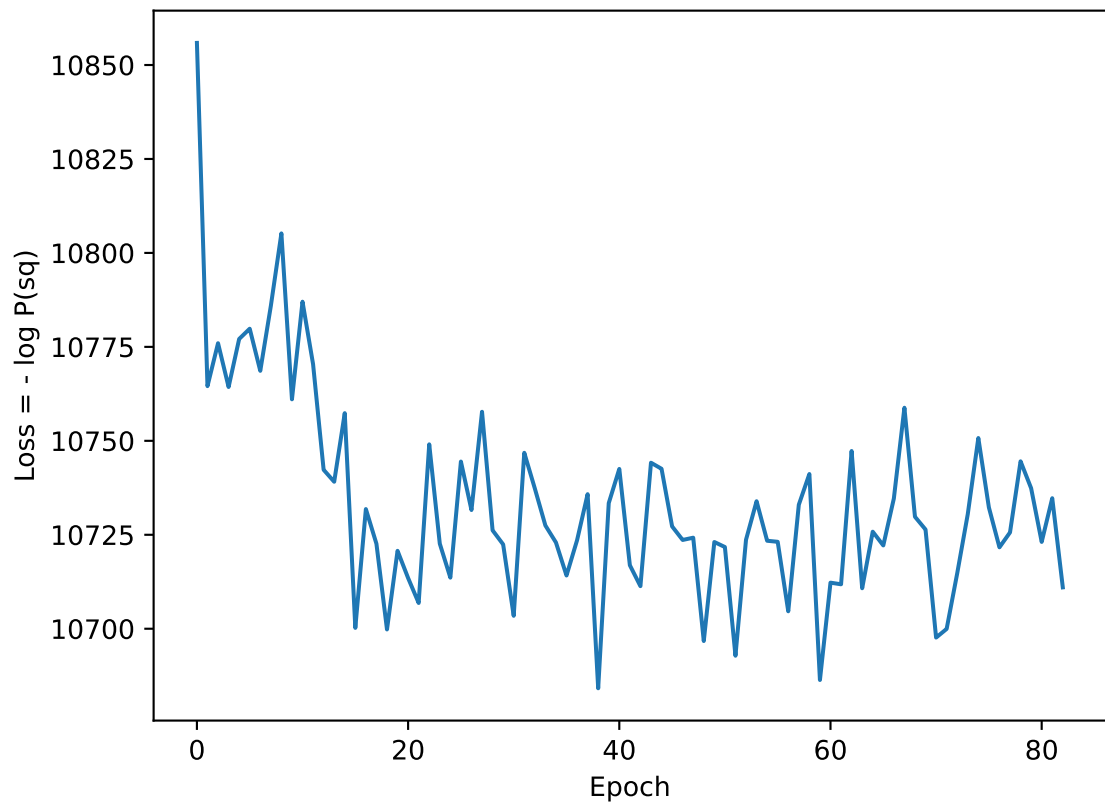

Supplement: Supplement 1 [file media-1.gz › supplemental_material/experiments/g6_optimize_param_conus_rnabench_RNaseP_uniform_shuffle/losses_i83.pdf]

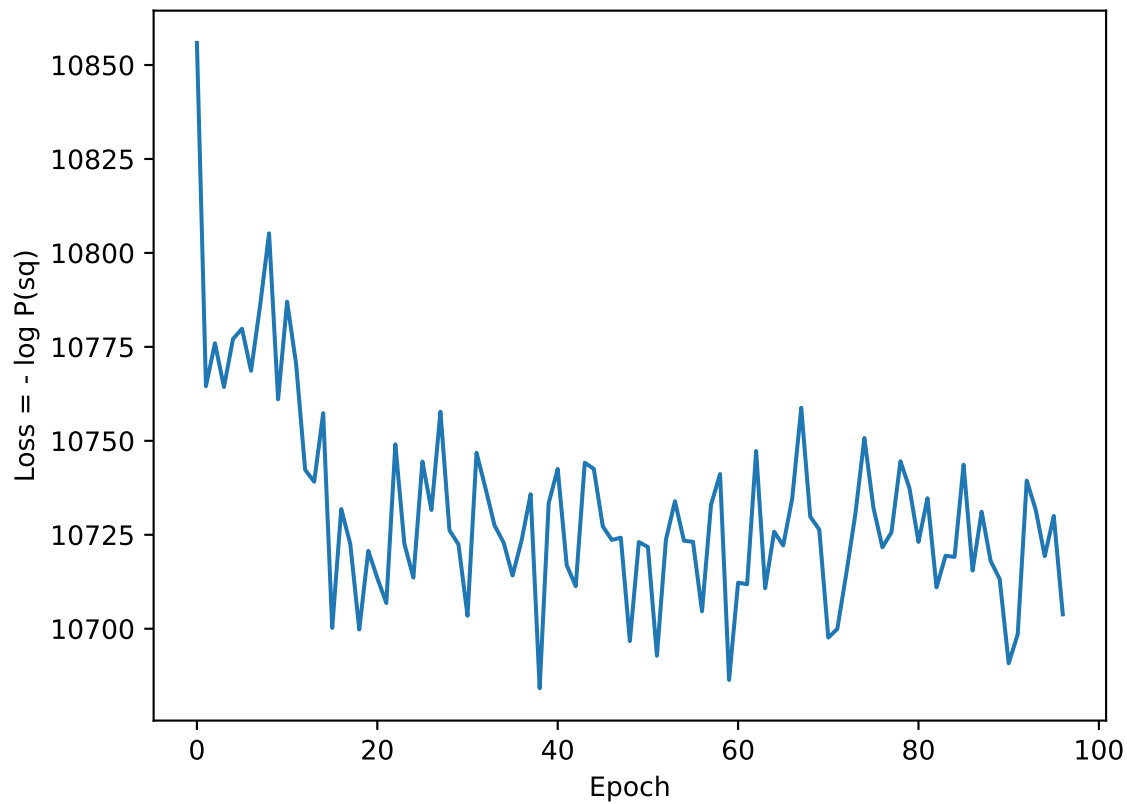

Supplement: Supplement 1 [file media-1.gz › supplemental_material/experiments/g6_optimize_param_conus_rnabench_RNaseP_uniform_shuffle/losses_i97.pdf]

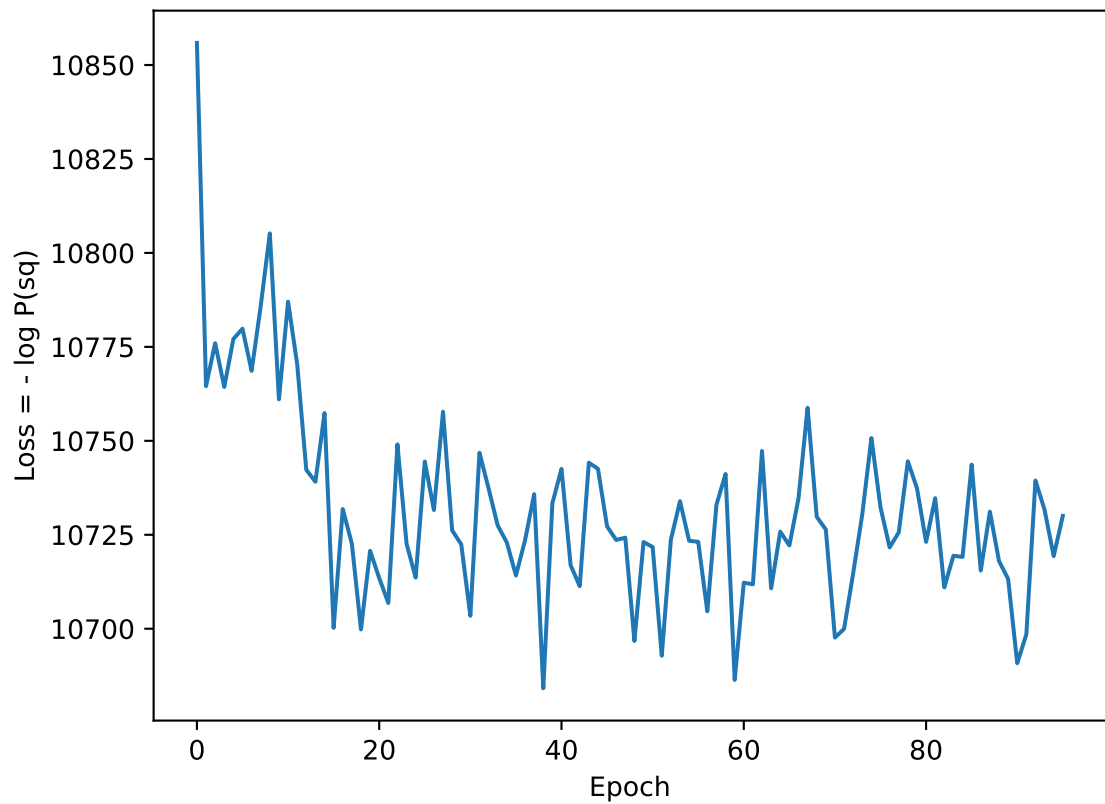

Supplement: Supplement 1 [file media-1.gz › supplemental_material/experiments/g6_optimize_param_conus_rnabench_RNaseP_uniform_shuffle/losses_i96.pdf]

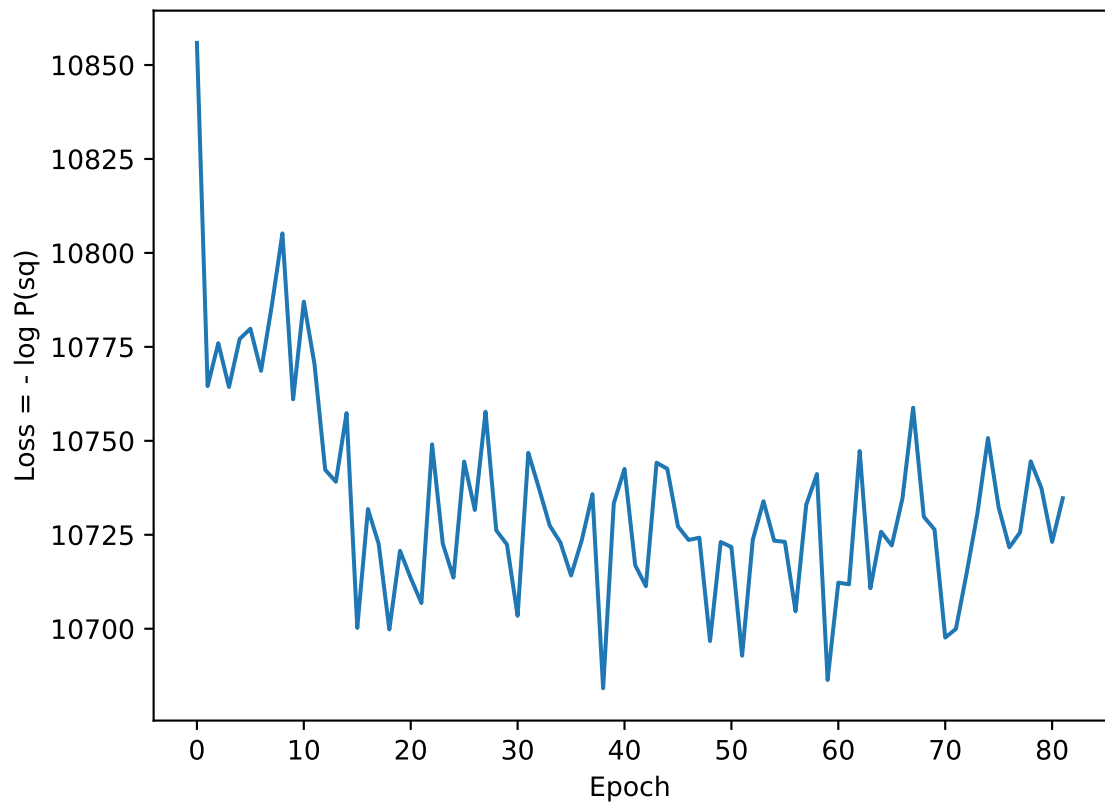

Supplement: Supplement 1 [file media-1.gz › supplemental_material/experiments/g6_optimize_param_conus_rnabench_RNaseP_uniform_shuffle/losses_i82.pdf]

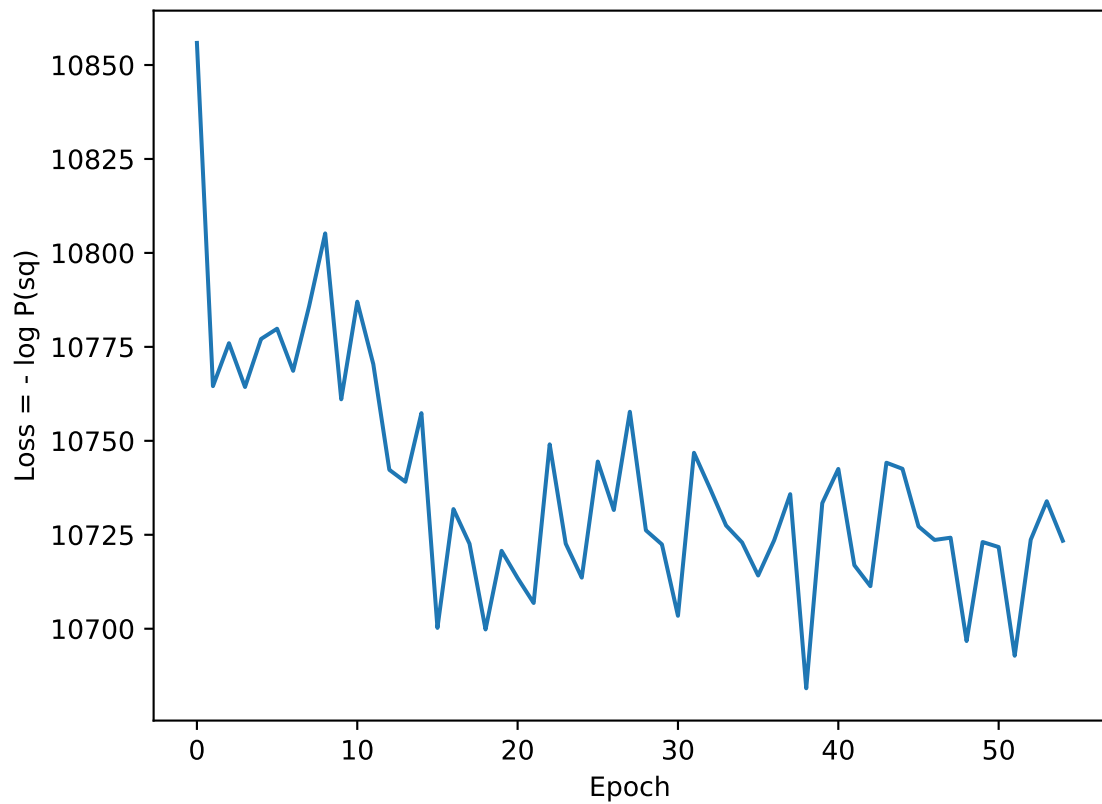

Supplement: Supplement 1 [file media-1.gz › supplemental_material/experiments/g6_optimize_param_conus_rnabench_RNaseP_uniform_shuffle/losses_i55.pdf]

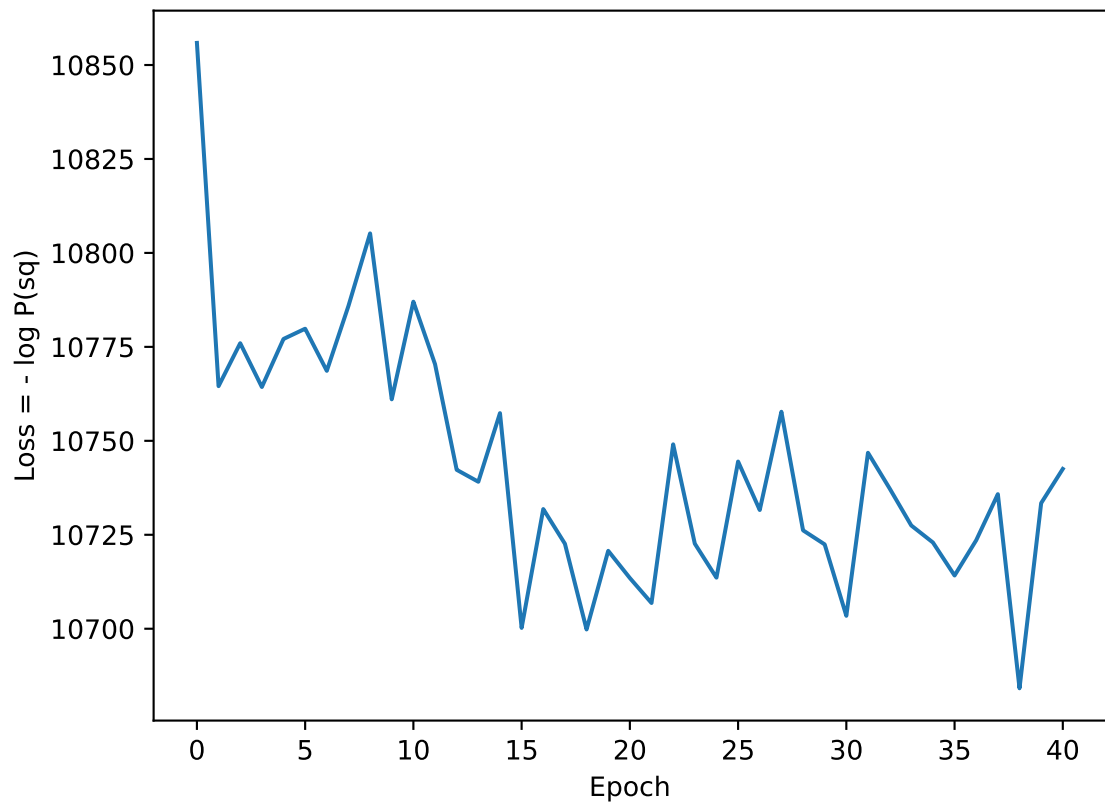

Supplement: Supplement 1 [file media-1.gz › supplemental_material/experiments/g6_optimize_param_conus_rnabench_RNaseP_uniform_shuffle/losses_i41.pdf]

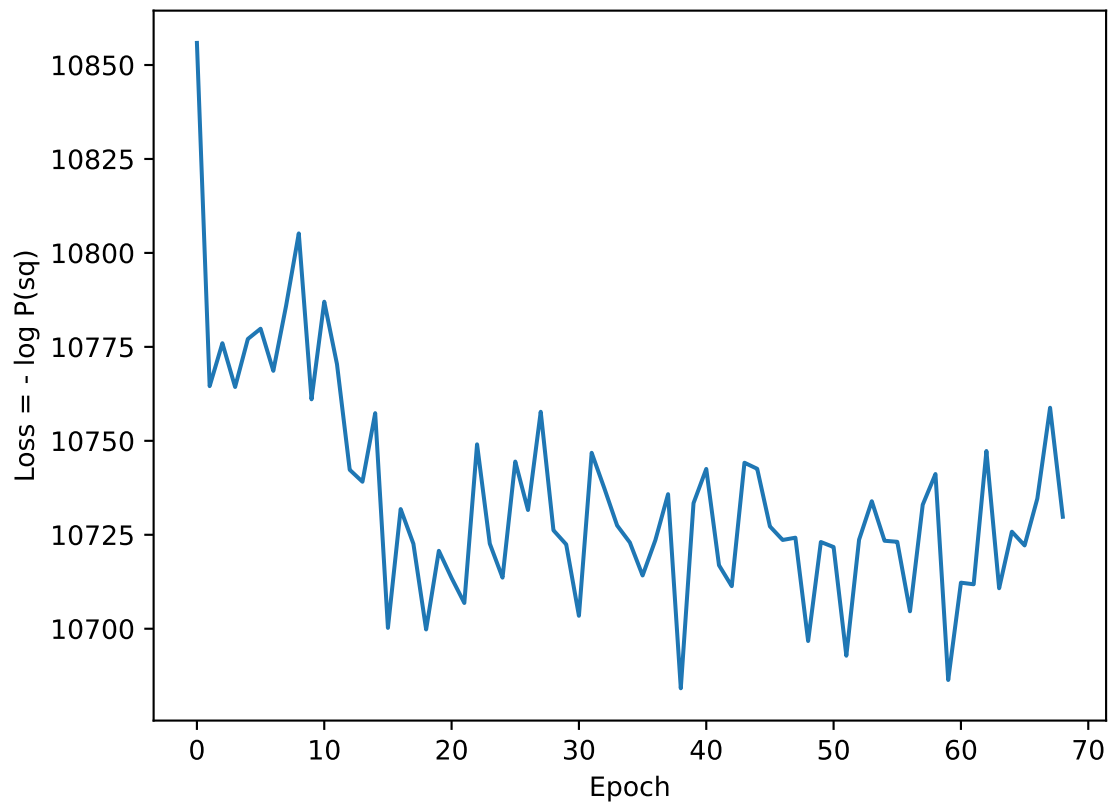

Supplement: Supplement 1 [file media-1.gz › supplemental_material/experiments/g6_optimize_param_conus_rnabench_RNaseP_uniform_shuffle/losses_i69.pdf]

# G6 grammar TORNADO\_conus\_rnabench\_RNaseP\_g6

Pair Probabilities  $P(ab)$  [ $\sum_{ab} P(ab) = 1$ ]  $a, b = \{A, C, G, U\}$

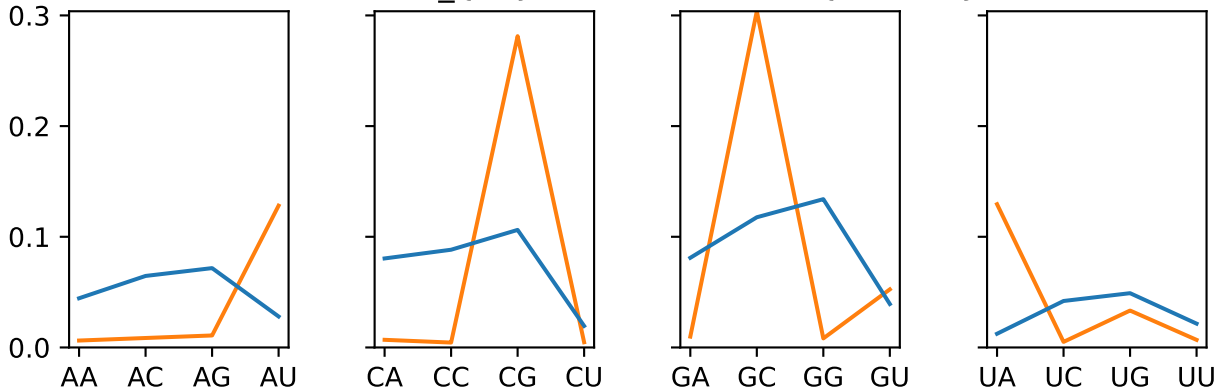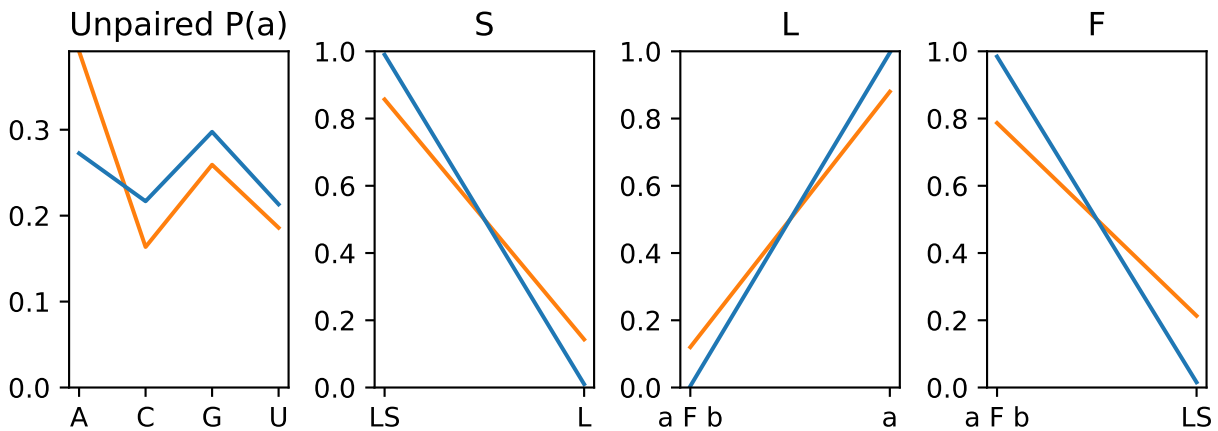

Supplement: Supplement 1 [file media-1.gz › supplemental_material/experiments/g6_optimize_param_conus_rnabench_RNaseP_uniform_shuffle/g6_params_i60.pdf]

# G6 grammar TORNADO\_conus\_rnabench\_RNaseP\_g6

Pair Probabilities  $P(ab)$  [ $\sum_{ab} P(ab) = 1$ ]  $a, b = \{A, C, G, U\}$

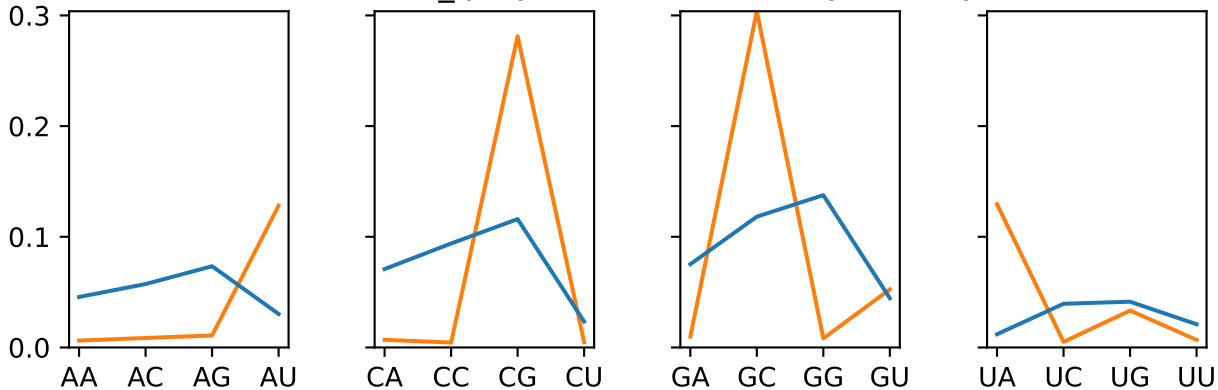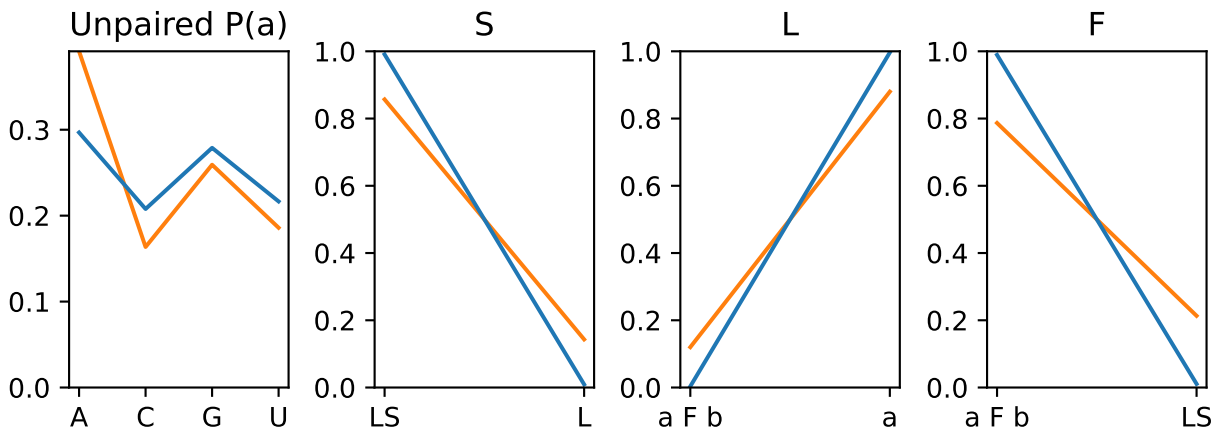

Supplement: Supplement 1 [file media-1.gz › supplemental_material/experiments/g6_optimize_param_conus_rnabench_RNaseP_uniform_shuffle/g6_params_i74.pdf]

# G6 grammar TORNADO\_conus\_rnabench\_RNaseP\_g6

Pair Probabilities  $P(ab)$  [ $\sum_{ab} P(ab) = 1$ ]  $a, b = \{A, C, G, U\}$

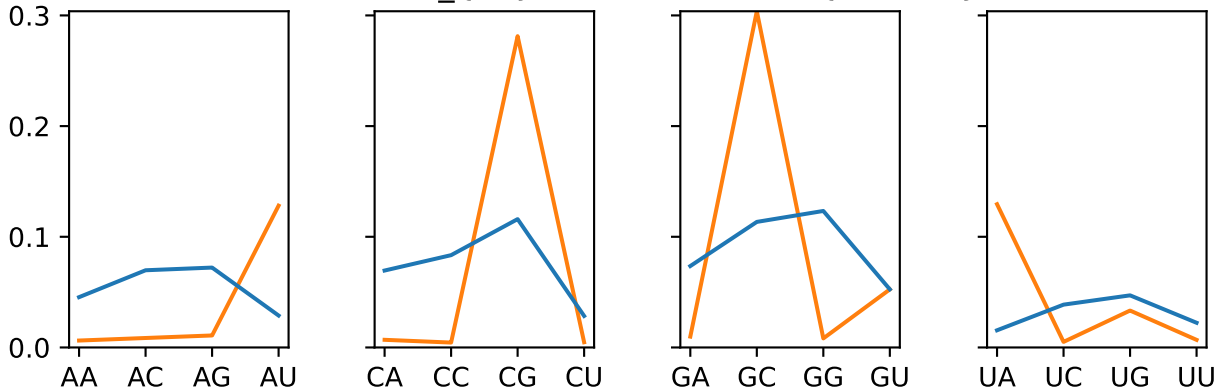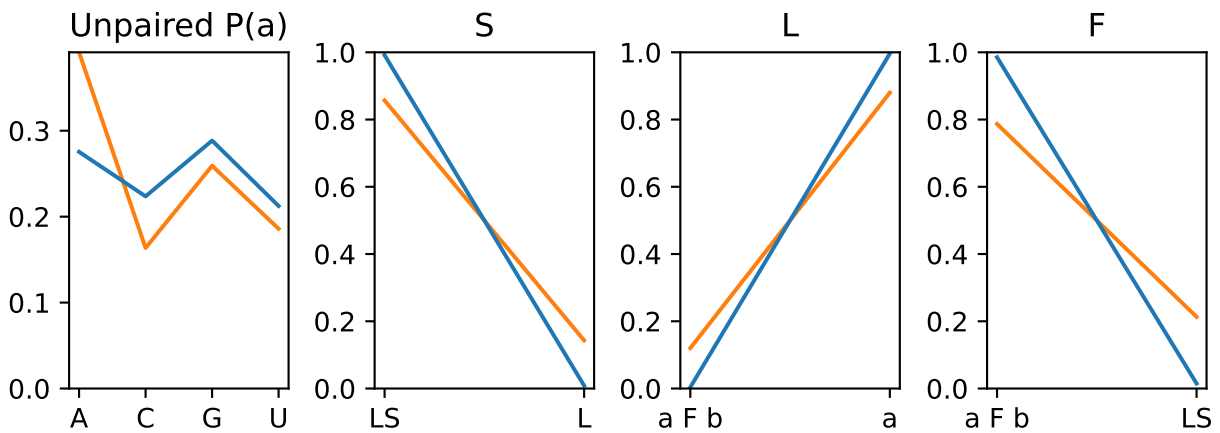

Supplement: Supplement 1 [file media-1.gz › supplemental_material/experiments/g6_optimize_param_conus_rnabench_RNaseP_uniform_shuffle/g6_params_i48.pdf]

# G6 grammar TORNADO\_conus\_rnabench\_RNaseP\_g6

Pair Probabilities  $P(ab)$  [ $\sum_{ab} P(ab) = 1$ ]  $a, b = \{A, C, G, U\}$

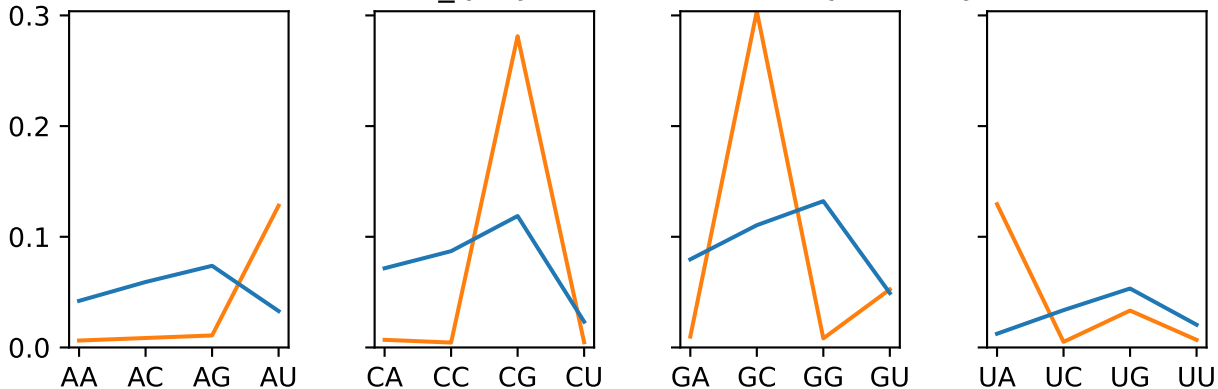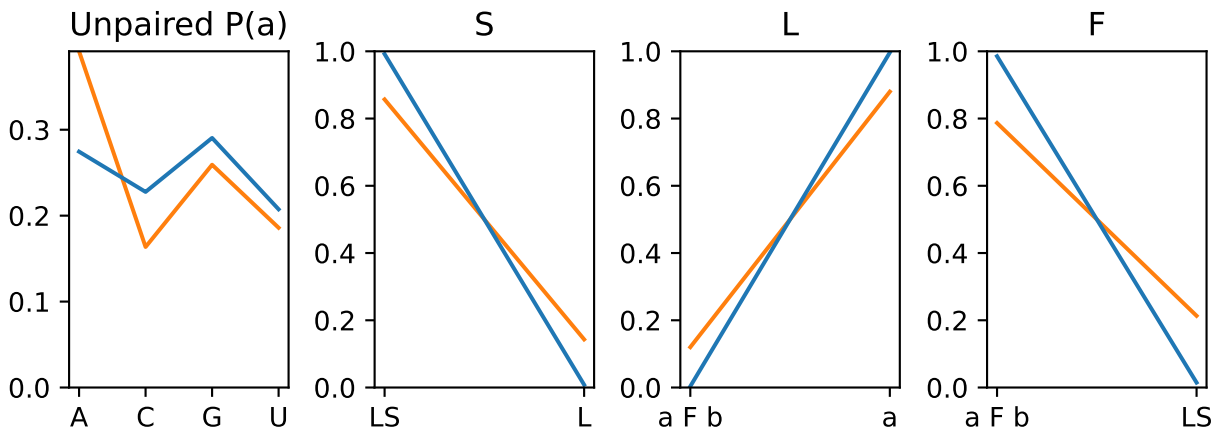

Supplement: Supplement 1 [file media-1.gz › supplemental_material/experiments/g6_optimize_param_conus_rnabench_RNaseP_uniform_shuffle/g6_params_i70.pdf]

# G6 grammar TORNADO\_conus\_rnabench\_RNaseP\_g6

Pair Probabilities  $P(ab)$  [ $\sum_{ab} P(ab) = 1$ ]  $a, b = \{A, C, G, U\}$

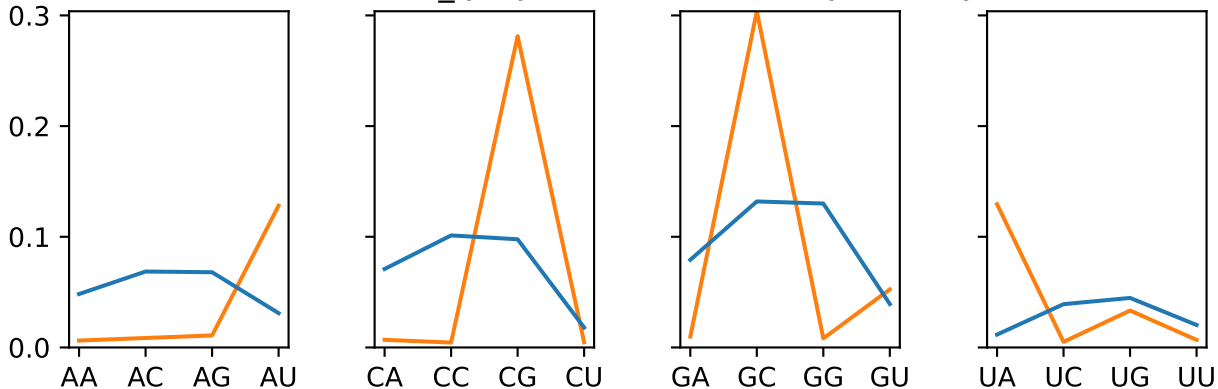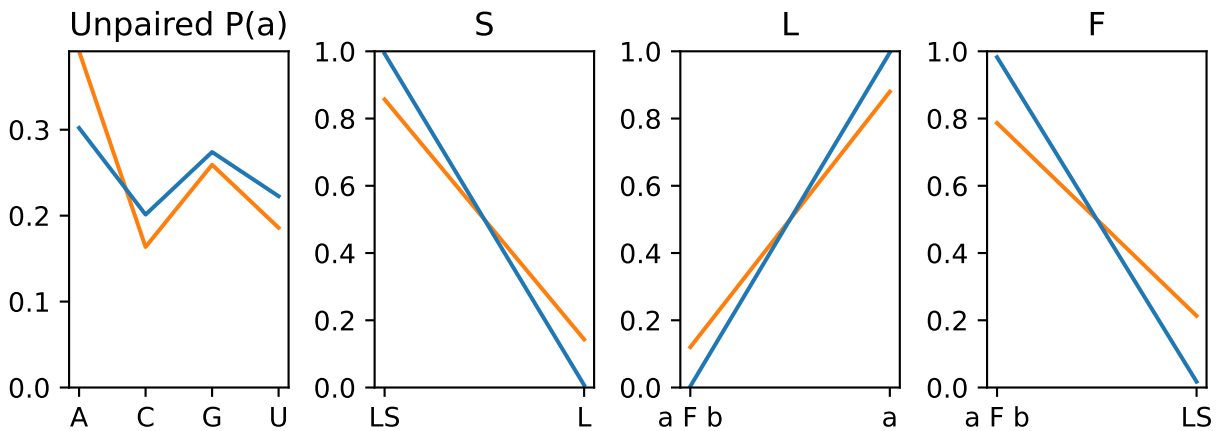

Supplement: Supplement 1 [file media-1.gz › supplemental_material/experiments/g6_optimize_param_conus_rnabench_RNaseP_uniform_shuffle/g6_params_i64.pdf]
