# Supplementary material for: What does it take to learn the rules of RNA base pairing? A lot less than you may think
Source: bioRxiv. 2025 Aug 2:2025.07.31.668042. Preprint. [Version 1] doi: 10.1101/2025.07.31.668042 (PMC12324431; doi:10.1101/2025.07.31.668042)
Supplement: 1 [file NIHPP2025.07.31.668042V1-supplement-1.pdf]

bioRxiv preprint doi: https://doi.org/10.1101/2025.07.31.668042; this version posted August 2, 2025. The copyright holder for this preprint (which was not certified by peer review) is the author/funder, who has granted bioRxiv a license to display the preprint in perpetuity. It is made available under aCC-BY 4.0 International license.

# Extended Data Figures

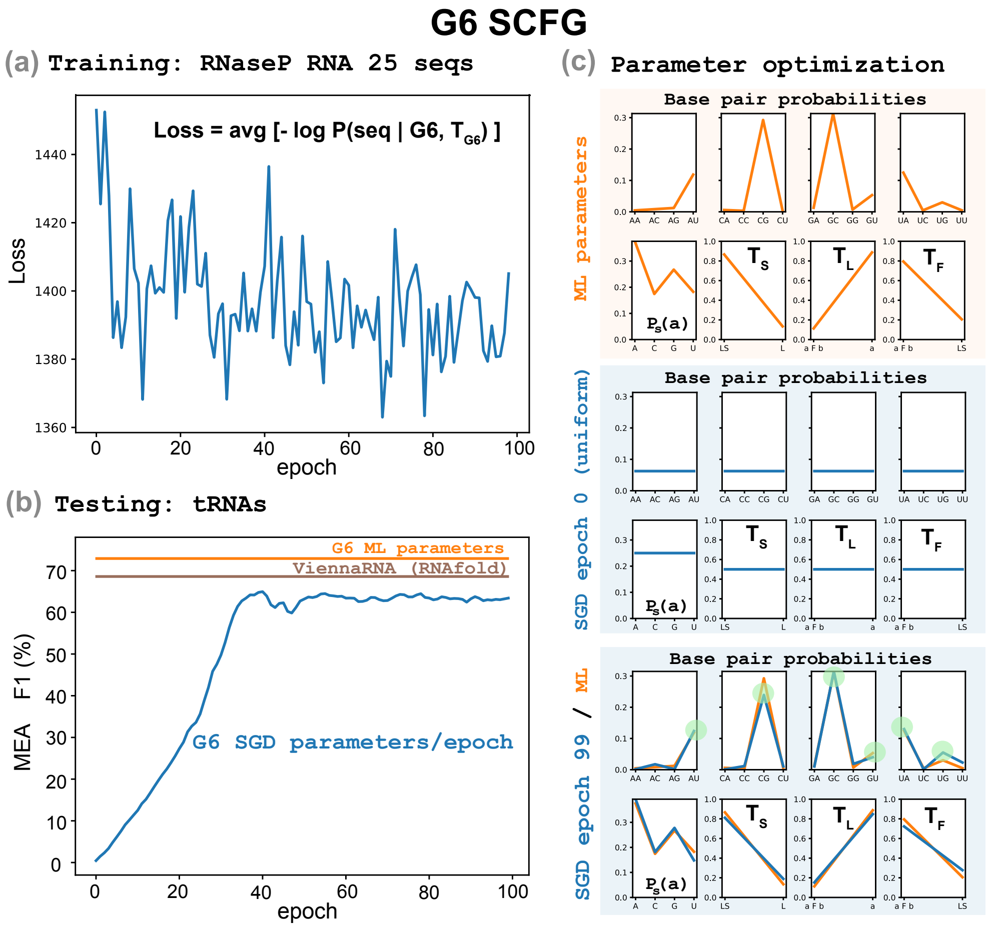


Figure S1: **G6 SGD training on 25 RNaseP RNA sequences.** We tested performance of our SGD optimization algorithm on a random subsample of 25 RNaseP RNA sequences. Legends for the different sections are similar to those in Figure 2.
